# Prophylactic Oropharyngeal Surfactant for Preterm Newborns at Birth: A Randomized Clinical Trial

**DOI:** 10.1001/jamapediatrics.2023.5082

**Published:** 2023-12-11

**Authors:** Madeleine C. Murphy, Jan Miletin, Claus Klingenberg, Hans Jørgen Guthe, Vincent Rigo, Richard Plavka, Kajsa Bohlin, Almerinda Barroso Pereira, Tomáš Juren, Ekele Alih, Marie Galligan, Colm P. F. O’Donnell

**Affiliations:** 1 National Maternity Hospital, Dublin, Ireland; 2 School of Medicine, University College Dublin, Dublin, Ireland; 3 National Children’s Research Centre, Dublin, Ireland; 4 Coombe Women and Infants University Hospital, Dublin, Ireland; 5 Paediatric Research Group, Faculty of Health Sciences, UiT–The Arctic University of Norway, Tromsø, Norway; 6 Department of Pediatrics and Adolescence Medicine, University Hospital of North Norway, Tromsø, Norway; 7 Haukeland University Hospital, Bergen, Norway; 8 Centre Hospitalier Universitaire de Liège, Liège, Belgium; 9 Charles University, Prague, Czech Republic; 10 Karolinska University Hospital, Stockholm, Sweden; 11 Karolinska Institutet, Stockholm, Sweden; 12 Hospital de Braga, Braga, Portugal; 13 University Hospital Brno, Brno, Czech Republic; 14 Clinical Research Centre, School of Medicine, University College Dublin, Dublin, Ireland

## Abstract

**Importance:**

Preterm newborns at risk of respiratory distress syndrome are supported with continuous positive airway pressure (CPAP). Many newborns worsen despite CPAP and are intubated for surfactant administration, an effective therapy for treatment of respiratory distress syndrome. Endotracheal intubation is associated with adverse effects. Pharyngeal administration of surfactant to preterm animals and humans has been reported as an alternative.

**Objective:**

To assess whether giving prophylactic oropharyngeal surfactant to preterm newborns at birth would reduce the rate of intubation for respiratory failure.

**Design, Setting, and Participants:**

This unblinded, parallel-group randomized clinical trial (Prophylactic Oropharyngeal Surfactant for Preterm Infants [POPART]) was conducted from December 17, 2017, to September 11, 2020, at 9 tertiary neonatal intensive care units in 6 European countries. Newborns born before 29 weeks of gestation without severe congenital anomalies, for whom intensive care was planned, were eligible for inclusion. The data were analyzed from July 27, 2022, to June 20, 2023.

**Intervention:**

Newborns were randomly assigned to receive oropharyngeal surfactant at birth in addition to CPAP or CPAP alone. Randomization was stratified by center and gestational age (GA).

**Main Outcomes and Measures:**

The primary outcome was intubation in the delivery room for bradycardia and/or apnea or in the neonatal intensive care unit for prespecified respiratory failure criteria within 120 hours of birth. Caregivers were not masked to group assignment.

**Results:**

Among 251 participants (mean [SD] GA, 26 [1.5] weeks) who were well matched at study entry, 126 (69 [54.8%] male) with a mean (SD) birth weight of 858 (261) grams were assigned to the oropharyngeal surfactant group, and 125 (63 [50.4%] male) with a mean (SD) birth weight of 829 (253) grams were assigned to the control group. The proportion of newborns intubated within 120 hours was not different between the groups (80 [63.5%) in the oropharyngeal surfactant group and 81 [64.8%] in the control group; relative risk, 0.98 [95% CI, 0.81-1.18]). More newborns assigned to the oropharyngeal surfactant group were diagnosed with and treated for pneumothorax (21 [16.6%] vs 8 [6.4%]; *P* = .04).

**Conclusions and Relevance:**

This randomized clinical trial found that administration of prophylactic oropharyngeal surfactant to newborns born before 29 weeks’ GA did not reduce the rate of intubation in the first 120 hours of life. These findings suggest that administration of surfactant into the oropharynx immediately after birth in addition to CPAP should not be routinely used.

**Trial Registration:**

EudraCT: 2016-004198-41

## Key Points

**Question:**

Does giving prophylactic oropharyngeal surfactant at birth in addition to continuous positive airway pressure (CPAP) to preterm newborns result in fewer newborns being intubated and receiving ventilation for respiratory failure within 120 hours of birth?

**Findings:**

In this randomized clinical trial of 251 newborns born before 29 weeks of gestation, giving prophylactic oropharyngeal surfactant at birth did not result in fewer newborns being intubated for respiratory failure within 120 hours of birth.

**Meaning:**

These findings suggest that because prophylactic surfactant at birth did not reduce the rate of intubation among preterm newborns in the first 120 hours of life, the treatment should not be routinely used.

## Teaser

This randomized clinical trial assesses whether administering prophylactic oropharyngeal surfactant at birth in addition to continuous positive airway pressure to preterm newborns results in fewer newborns being intubated for respiratory failure in the first 120 hours of life.

## Introduction

Neonatal respiratory distress syndrome (RDS) is characterized by a relative lack of surfactant.^1^ Exogenous surfactant,^2^ given via an endotracheal tube (ETT), revolutionized the care of preterm newborns in the 1990s.^3,4,5^ However, laryngoscopy, intubation, and mechanical ventilation are associated with adverse outcomes.^6,7,8^ Treating preterm newborns with continuous positive airway pressure (CPAP) and reserving intubation, surfactant, and ventilation for newborns whose breathing worsens despite CPAP has been associated with better results than routinely intubating this population for surfactant administration.^9^ Randomized clinical trials^10,11,12^ that compared CPAP with intubation reported rates of mechanical ventilation in the days after birth of 40% to 60% among preterm newborns on CPAP. A thin catheter surfactant technique was reported to reduce the rate of mechanical ventilation from 46% to 28% among newborns born 26 weeks’ to 28 weeks’ gestation.^13^

A desire for preterm newborns to have the benefits of surfactant without the adverse effects of intubation and ventilation has prompted the search for alternative methods of administering surfactant.^14^ Using the intubation-surfactant-extubation (INSURE) technique, newborns treated on CPAP are intubated with an ETT for surfactant administration with the intention to extubate them promptly. With this approach, laryngoscopy and intubation are not avoided, and a proportion of babies are not successfully extubated. Surfactant may also be given to newborns treated with nasal CPAP through a thin catheter inserted into the trachea.^13,15,16,17^ While this approach largely avoids the use of positive pressure ventilation, laryngoscopy is not avoided. A meta-analysis^18^ reported that surfactant administration via a thin catheter technique was associated with fewer deaths or bronchopulmonary dysplasia (BPD), less intubation within 72 hours, and reduced in-hospital mortality. More recently, Dargaville et al^19^ studied the effect of giving thin catheter surfactant to newborns of 25 to 28 weeks’ gestational age (GA) with RDS receiving more than 30% oxygen on CPAP and found that it did not increase their survival without BPD when compared with those treated on CPAP alone. Nebulized surfactant has had limited success.^20,21,22,23,24,25^ In a recent randomized clinical trial,^24^ prophylactic nebulized surfactant in addition to CPAP in the delivery room did not improve aeration or clinical outcomes among newborns born between 26 and 32 weeks’ GA compared with CPAP alone. Supraglottic airways have been used to deliver surfactant^26,27,28,29^; however, currently, to our knowledge, no devices are available for use among newborns with extremely low birth weight who are most likely to be diagnosed with and treated for RDS.

Surfactant administration into the pharynx is a simple technique that does not require new or expensive equipment and avoids laryngoscopy. Preterm rabbits that received pharyngeal surfactant and allowed a period of spontaneous breathing showed higher lung-thorax compliance compared with those ventilated, and approximately half of the surfactant reached the lungs.^30^ Administration of surfactant into the pharynx of human newborns has been described in a randomized clinical trial^4^ and in cohort studies.^31,32^ In 1 randomized clinical trial that compared artificial surfactant with saline,^4^ preterm newborns received the first dose via the oropharynx, with subsequent doses given via ETT if the newborn was intubated. In this study, surfactant significantly reduced mortality and the need for respiratory support, but the outcomes of newborns who received only pharyngeal surfactant were not reported. A systematic review concluded that randomized clinical trials are needed to assess the efficacy of pharyngeal surfactant.^33^

In the present study, we assessed whether giving preterm newborns surfactant into their oropharynx at birth in addition to CPAP compared with CPAP alone would reduce the rate of intubation in the first 120 hours of life.

## Methods

### Trial Design

We conducted Prophylactic Oropharyngeal Surfactant for Preterm Infants: A Randomised Trial (POPART), an investigator-led, unblinded, parallel-group randomized clinical trial, at 9 university hospitals in 6 European countries: National Maternity Hospital, Dublin, Ireland; Coombe Women and Infants University Hospital, Dublin, Ireland; University Hospital of North Norway, Tromsø, Norway; Haukeland University Hospital, Bergen, Norway; Charles University, Prague, Czech Republic; University Hospital Brno, Brno, Czech Republic; Centre Hospitalier Universitaire, Liège, Belgium; Karolinska University Hospital, Stockholm, Sweden; and Hospital de Braga, Braga, Portugal, between December 17, 2017, and September 11, 2020. The trial was approved by research ethics committees and competent authorities in each participating country.^34^ The Clinical Research Centre at University College Dublin was the study sponsor. Written informed consent for participation was obtained from parents or guardians before the newborns’ birth. Data were analyzed from July 27, 2022, to June 20, 2023. This study followed the Consolidated Standards of Reporting Trials (CONSORT) reporting guideline.^35^

The trial protocol is provided in Supplement 1. A nonsubstantial protocol amendment, in relation to the definition of the end of the trial and close-out activities at different sites, was issued following publication of the protocol.^34^ In addition, the published protocol includes a further description of the treatment plan at the time of first intubation, which relates to the primary outcome.

### Participants

Newborns born before 29 weeks’ GA for whom intensive care was planned were eligible for inclusion. Newborns were ineligible if they had major congenital anomalies.

### Randomization and Blinding

Newborns were randomly assigned (1:1) before birth to receive oropharyngeal surfactant at birth in addition to CPAP or CPAP alone. Randomization was stratified by center and GA (<26 weeks and 26-28 weeks plus 6 days of gestation). Newborns of multiple gestations were randomized as individuals. An independent statistician (M.G.) prepared the schedule in permuted blocks of 4, 6, and 8 using a computer program. Group assignment was contained in sequentially numbered, sealed, opaque envelopes that were opened immediately before birth. Neither caregivers nor outcome assessors were masked to group assignment. The trial statistician (E.A.) was blinded for data analysis.

### Oropharyngeal Surfactant Intervention and Standard Care Control Group

Poractant alfa (Chiesi Farmaceutici) is a natural surfactant approved for endotracheal use for the prevention and treatment of RDS. Doses of 100 mg/kg to 200 mg/kg are recommended for prophylaxis and 200 mg/kg for treatment of established RDS. It is commercially available in vials that contain 120 mg or 240 mg of surfactant. Participants were not weighed prior to enrollment; newborns less than 26 weeks’ GA received a 120-mg vial, and newborns 26 weeks to 28 weeks plus 6 days of gestation received a 240-mg vial. The surfactant was warmed before being prepared in a sterile syringe when birth was imminent. A flexible thin catheter, 5 cm long, was attached to the syringe, and the surfactant was instilled into the oropharynx as soon as possible after delivery. Newborns were not suctioned and did not receive mask CPAP or positive pressure ventilation beforehand. The surfactant was to be given from 30 seconds to 60 seconds, ideally before the umbilical cord was clamped. Newborns were then treated on CPAP. Newborns randomly assigned to the control group did not have anything instilled into their oropharynx and were stabilized on CPAP in the delivery room.

### Clinical Treatment

After the initial intervention, newborns received delivery room care according to recommendations of the International Liaison Committee on Resuscitation regardless of their group assignment.^36^ Newborns were intubated in the delivery room for persistent apnea and/or bradycardia despite mask positive pressure ventilation. Newborns were not intubated in the delivery room solely for surfactant administration. After admission to the neonatal intensive care unit (NICU), enrolled newborns were intubated if they met predetermined criteria for respiratory failure. Frequency of blood gas measurements and other aspects of care were at the discretion of the treating physicians. The dosage of ETT surfactant was not affected by group assignment. If a newborn was determined to need ETT surfactant following oropharyngeal administration, it was provided at the standard initial dose of 200 mg/kg via ETT; the technique of administration (ie, ETT, INSURE, or thin catheter) was at the discretion of treating physicians.

### Outcome Measures and Data Management

The primary outcome was endotracheal intubation for respiratory failure within 120 hours of birth. Newborns were intubated for persistent apnea and/or bradycardia (heart rate <100 beats per minute) despite mask ventilation in the delivery room or for respiratory failure in the NICU, defined as 2 or more of the following signs and blood test results: (1) worsening clinical signs (tachypnea; grunting; or subcostal, intercostal, and/or sternal recession), (2) acidosis (pH <7.2 on 2 blood gas measurements [arterial or capillary] ≥30 minutes apart), (3) hypoxemia (fraction of inspired oxygen >0.4 to keep oxygen saturation ≥90% for >30 minutes), (4) hypercarbia (partial pressure of carbon dioxide >9.0 kPa on 2 blood gases [arterial or capillary] ≥30 minutes apart), and (5) recurrent apnea treated with mask ventilation. Newborns reached the primary outcome if they were intubated with an ETT for mechanical ventilation, intubated with an ETT for surfactant administration and mechanical ventilation, or intubated with an ETT for surfactant administration and extubated to CPAP (INSURE) or if they had laryngoscope-guided thin catheter surfactant administration.

We collected data on the following secondary outcomes: intubation in the delivery room, number of attempts taken to intubate in the delivery room, chest compressions in the delivery room, epinephrine administration in the delivery room, NICU intubation, endotracheal surfactant use before death or hospital discharge (number of doses and total dose), RDS, pneumothorax and treatment with needle aspiration or chest drain insertion, pulmonary hemorrhage, mechanical ventilation, days of mechanical ventilation, use of postnatal corticosteroids, duration of respiratory support, BPD (supplemental oxygen at day 28), chronic lung disease of prematurity (supplemental oxygen treatment at 36 weeks’ corrected GA), physiological BPD determined by a physiological oxygen reduction test, medical and surgical treatment for a patent ductus arteriosus, necrotizing enterocolitis (Bell stage 2 or higher), intraventricular hemorrhage (any and severe [grade 3 or 4]), cystic periventricular leukomalacia, retinopathy of prematurity treated with laser or intravitreal injections, death before discharge, survival without BPD at discharge, survival without chronic lung disease at discharge, duration of hospitalization, and use of home oxygen. Data were collected from the patient’s medical records, recorded on a worksheet, and transferred to an electronic case report form for storage in a secure, password-protected, electronic database.^37^

### Safety Monitoring

Adverse events were reported in accordance with Good Clinical Practice guidelines. A data safety monitoring board met at 6-month intervals to perform safety surveillance and interim analyses. Interim analyses were carried out after 50% of participants completed the study. The data safety monitoring board could have recommended early termination of the trial due to efficacy or futility or for safety concerns.

### Statistical Analysis

The sample size was calculated in G*Power, version 3.1.9.2 (Heinrich Heine Universität Düsseldorf) based on a 2-sided, 2-proportion *z* test. We assumed a rate of intubation within 120 hours of 46% for newborns treated with CPAP. We hypothesized that oropharyngeal surfactant would reduce the need for intubation within 120 hours and that a sample of 250 newborns (125 per group) would give a statistical power of 80% at a significance level of 5% to demonstrate a reduction in the proportion intubated from 46% to 28% (absolute reduction of 18%; relative reduction of 39%) adjusted for an anticipated death rate before 120 hours of 10%.

The primary outcome was summarized per group. The ratios of relative risk (RR) were reported with 95% CIs. A 2-sided, 2-proportion *z* test was carried out to investigate whether the rate of endotracheal intubation differed between intervention and standard of care. The main analysis of the primary end point was conducted on the full analysis set, used data on patients up to 120 hours of life while alive, and assumed that newborns intubated within 120 hours met the protocol-defined criteria for respiratory failure. A number of predefined sensitivity analyses are described in eAppendix 1 in Supplement 2.

Categorical outcomes were summarized per treatment group, with between-group differences expressed as relative risk with 95% CIs. A 2-sided, 2-proportion *z* test was carried out for each categorical outcome to investigate whether the proportion differed between intervention and standard of care. The effect of the intervention on numeric secondary end points was quantified as a difference in medians, with 95% bootstrap CIs. A Mann-Whitney *U* test was used to test for a difference in the distribution of the end point between treatment groups. For handling missing data, we followed a published flowchart.^38^ For end points with less than 5% missingness, a complete case analysis was performed with a sensitivity analysis using best-worst and worst-best case analyses. For missingness of 5% or more, multiple imputation was carried out during analysis.

Subgroups of interest included newborns of different GA strata (eg, <26 weeks and 26 weeks to 28 weeks plus 6 days of gestation) and newborns from different centers. The effect of the intervention on the primary end point was estimated per subgroup using relative risk and 95% CI. Log-binomial regression with an interaction effect between treatment and subgroup variables was used to determine whether the intervention effect differed by subgroup, where sample size permitted. A 2-sided *P* < .05 was considered significant.

## Results

Between December 2017 and September 2020, 252 newborns were randomized at 9 hospitals (132 at the National Maternity Hospital, 48 at the Coombe Women and Infants University Hospital, 30 at the Haukeland University Hospital, 13 at the Centre Hospitalier Universitaire, 11 at the Charles University, 9 at the University Hospital of North Norway, 6 at the Karolinska University Hospital, 2 at the Hospital de Braga, and 1 at the University Hospital Brno) (Figure). Of these participants, 223 (88.5%) were enrolled at 4 of the sites. At these 4 sites, 223 of 270 eligible newborns (82.6%) born during the study period were enrolled. Consent for participation was sought and obtained for many newborns who were born at or after 29 weeks’ GA and were ultimately ineligible. One newborn who was randomized was diagnosed with esophageal atresia shortly after birth and was excluded. We included 251 newborns (mean [SD] GA, 26 [1.5] weeks) in our final analysis, of whom 126 (57 [45.2%] female and 69 [54.8%] male) were assigned to oropharyngeal surfactant at birth in addition to CPAP and 125 (62 [49.6%] female and 63 [50.4%] male) to CPAP alone. Sixty percent of participants were enrolled outside regular daytime working hours.

**Figure.**
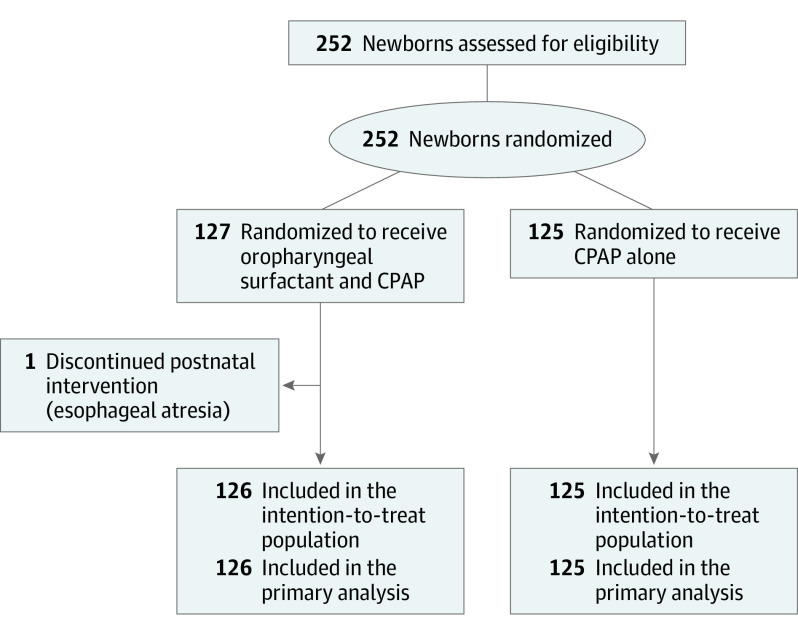
CONSORT Diagram CONSORT indicates Consolidated Standards of Reporting Trials and CPAP, continuous positive airway pressure.

The characteristics of the 2 groups were similar at study entry (Table 1); newborns assigned to the oropharyngeal surfactant group had a mean (SD) birth weight of 858 (261) grams, and those assigned to the control group had a mean (SD) birth weight of 829 (253) grams. Ten newborns weighed less than 500 g at birth, and 3 newborns weighed less than 400 grams. The median (IQR) dose of oropharyngeal surfactant was 212 mg/kg (173-271 mg/kg) for the intervention group overall (<26 weeks’ GA: 172 mg/kg [153-207 mg/kg]; ≥26 weeks’ GA: 246 mg/kg [203-294 mg/kg]). In the intervention group, all newborns received a dose of more than 100 mg/kg, and 76 (60.3%) received a dose of more than 200 mg/kg.

**Table 1.** Baseline Characteristics of Mothers and Newborns at Study Entry

Characteristic	Participants, No. (%)	
	Oropharyngeal surfactant group (n = 126)	Control group (n = 125)
**Mothers**		
Exposure to ANS	126 (100.0)	125 (100.0)
Completed ANS	116 (92.1)	107 (85.6)
Cesarean delivery	84 (66.6)	78 (62.4)
PPROM	55 (43.7)	51 (40.8)
Duration of PPROM, median (IQR), h	123 (49-412)	62 (25-173)
**Newborns**		
Gestational age, mean (SD), wk	26 (1.5)	26 (1.5)
Gestational age <26 wk	48 (38.1)	44 (35.2)
Birth weight, mean (SD), g	858 (261)	829 (253)
Sex		
Female	57 (45.2)	62 (49.6)
Male	69 (54.8)	63 (50.4)
Multiple birth	44 (34.9)	45 (36.0)
Time of cord clamping, median (IQR), s	60 (50-70)	60 (40-60)
Apgar score at 5 min, mean (SD)^a^	8 (2)	8 (2)

Abbreviations: ANS, antenatal steroids; PPROM, preterm premature rupture of membranes.

^a^ Scores range from 0 to 10, with higher scores indicating greater physical well-being of newborn.

The proportion of newborns intubated by 120 hours was not different between groups (80 [63.5%] in the oropharyngeal surfactant group and 81 [64.8%] in the control group; RR, 0.98 [95% CI, 0.81-1.18]; *P* = .93) (Table 2). Treatment effect did not differ substantially by center. The rate of the primary outcome was higher in the younger GA stratum (oropharyngeal surfactant group: 85.4%; control group: 79.5%) compared with the older GA stratum (oropharyngeal surfactant group: 50.0%; control group: 56.8%) and did not differ between the groups in either stratum (younger GA: RR, 1.07 [95% CI, 0.89-1.30] vs older GA: RR, 0.88 [95% CI, 0.66-1.18]; *P* = .26).

**Table 2.** Primary Outcome by Treatment Group and Subgroup

Outcome	Newborns, No. (%)		Relative risk (95% CI)	*P* value
	Oropharyngeal surfactant group (n = 126)	Control group (n = 125)		
Intubation within 120 h of life	80 (63.5)	81 (64.8)	0.98 (0.81-1.18)	.93^a^
Gestational age <26 wk	41 of 48 (85.4)	35 of 44 (79.5)	1.07 (0.89-1.30)	.26^b^
Gestational age 26-28 wk plus 6 d	39 of 78 (50.0)	46 of 81 (56.8)	0.88 (0.66-1.18)	

^a^ *P* value is from a 2-sided *z* test.

^b^ *P* value corresponds to the interaction effect between gestational age and treatment group in a log-binomial regression.

Among the 80 newborns randomized to oropharyngeal surfactant and who were intubated, the plan at the time of first intubation was intubation and continued ventilation for 63 newborns (78.8%), INSURE for 12 (15.0%), less invasive surfactant administration for 2 (2.5%), and other techniques for 3 (3.8%). Among the 81 newborns randomized to CPAP alone and who were intubated, the plan at the time of first intubation was intubation and continued ventilation for 67 newborns (82.7%), INSURE for 6 (7.4%), less invasive surfactant administration for 6 (7.4%), and other techniques for 2 (2.5%) newborns.

In the delivery room, 5 newborns in the oropharyngeal surfactant group and 3 newborns in the control group were intubated in breach of the protocol, and the proportions did not differ by group assignment. A sensitivity analysis, in which participants intubated in breach of the protocol were considered not to have met the primary outcome, did not show any difference in the primary outcome between the groups (eTable 3 in Supplement 2). Further sensitivity analyses did not differ from the main analysis of the primary end point (eTables 1-8 and eFigure in Supplement 2). There were no statistically significant differences between the groups in the incidence of mechanical ventilation (78 newborns [61.9%] in the oropharyngeal surfactant group vs 84 [67.2%] in the control group; RR, 0.92 [95% CI, 0.76-1.11]; *P* = .46) and duration of mechanical ventilation (median [IQR] days in the oropharyngeal surfactant group, 1 [0-7] vs 2 [0-7] days in the control group; median [IQR] difference, −1.00 [−2.72 to −0.06] days; *P* = .47), intratracheal surfactant use (75 newborns [59.5%] in the oropharyngeal surfactant group vs 78 [62.4] in the control group; RR, 0.95 [95% CI, 0.78-1.16]; *P* = .74), BPD (73 of 105 newborns [70.0%] in the oropharyngeal surfactant group vs 74 of 107 [69.2%] in the control group; RR, 1.01 [95% CI, 0.84-1.21]; *P* > .99), chronic lung disease of prematurity (28 of 103 newborns [27.2%] in the oropharyngeal surfactant group vs 30 of 102 [29.4%] in the control group; RR, 0.92 [95% CI, 0.60-1.43]; *P* = .84), or postnatal corticosteroid use (29 newborns [23.0%] in the oropharyngeal surfactant group vs 30 [24.0] in the control group; RR, 0.96 [95% CI, 0.62-1.49]; *P* = .97) (Table 3).

**Table 3.** Secondary Outcomes

Outcome	Newborns, No. (%)		Relative risk or difference in median (95% CI)	*P* value
	Oropharyngeal surfactant group (n = 126)	Control group (n = 125)		
Treatment administered in the delivery room				
Intubation	28 (22.2)	38 (30.4)	0.73 (0.48 to 1.11)	.18
Attempts to intubate, median (IQR), No.^a^	1 (1 to 2)	1 (1 to 2)	0.00 (−0.76 to 1.24)	.40
Chest compressions	5 (4.0)	3 (2.4)	1.65 (0.45 to 6.16)	.73
Epinephrine	1 (<1.0)	0	NA	>.99
NICU rectal temperature, median (IQR), ° C^b^	36.3 (35.9 to 36.7)	36.5 (35.9 to 36.9)	−0.28 (−0.55 to 0.90)	.22
First intubation in the NICU	55 (43.7)	49 (39.2)	1.11 (0.83 to 1.50)	.55
Intratracheal surfactant	75 (59.5)	78 (62.4)	0.95 (0.78 to 1.16)	.74
Doses of postintervention intratracheal surfactant, median (IQR)^c^	2 (1 to 2)	1 (1 to 2)	1.00 (−0.44 to 2.57)	.19
Pneumothorax	21 (16.7)	8 (6.4)	2.60 (1.23 to 5.59)	.02
Pneumothorax treated with needle aspiration or chest drain	21 (16.7)	6 (4.8)	NA	.04
Pulmonary hemorrhage	6 (4.8)	5 (4.0)	1.19 (0.40 to 3.59)	>.99
Mechanical ventilation	78 (61.9)	84 (67.2)	0.92 (0.76 to 1.11)	.46
Duration of mechanical ventilation, No. (range), d	1 (0 to 7)	2 (0 to 7)	−1.00 (−2.72 to −0.06)	.47^d^
Postnatal corticosteroids	29 (23.0)	30 (24.0)	0.96 (0.62 to 1.49)	.97
Duration of respiratory support, median (IQR), d^e^	53 (27 to 73)	50 (26 to 70)	3.00 (−5.47 to 14.73)	.83
BPD^f^	73 of 105 (70.0)	74 of 107 (69.2)	1.01 (0.84 to 1.21)	>.99
CLD^g^	28 of 103 (27.2)	30 of 102 (29.4)	0.92 (0.60 to 1.43)	.84
Physiological BPD^h^	30 of 91 (33.0)	26 of 90 (28.9)	1.11 (0.72 to 1.70)	.61
Medical treatment for PDA	27 (21.4)	37 (29.6)	0.72 (0.47 to 1.11)	.18
Surgical treatment for PDA	2 (1.6)	2 (1.6)	0.99 (0.18 to 5.56)	>.99
Necrotizing enterocolitis	10 (7.9)	15 (12.0)	0.66 (0.31 to 1.39)	.39
IVH grade 3 or 4	7 (5.6)	9 (7.2)	0.80 (0.32 to 2.00)	.84
Cystic PVL	4 (3.2)	5 (4.0)	0.82 (0.24 to 2.76)	>.99
ROP treated with laser or intravitreal injections^i^	14 of 102 (13.7)	10 of 102 (9.8)	1.35 (0.61 to 3.01)	.46
Death before hospital discharge	23 (18.3)	22 (17.6)	1.03 (0.61 to 1.75)	>.99
Survival without BPD at hospital discharge	31 (24.6)	30 (24.0)	1.03 (0.66 to 1.58)	>.99
Survival without CLD at hospital discharge	71 (56.3)	72 (57.6)	0.97 (0.78 to 1.20)	.88
Duration of hospitalization, median (IQR), d	74 (54 to 93)	76 (53 to 89)	−2.00 (−9.22 to 3.60)	.87
Home oxygen therapy	5 (4.0)	10 (8.0)	0.48 (1.77 to 1.31)	.26

Abbreviations: BPD, bronchopulmonary dysplasia (supplemental oxygen at day 28); CLD, chronic lung disease (supplemental oxygen treatment at 36 weeks’ corrected gestational age); IVH, intraventricular hemorrhage; NA, not applicable; NICU, neonatal intensive care unit; PDA, patent ductus arteriosus; PVL, periventricular leukomalacia; ROP, retinopathy of prematurity.

^a^ Analyzed for newborns intubated in the delivery room only (n = 28 in the intervention group; n = 38 in the control group).

^b^ Missing data frequency was greater than 40% and hence in line with the statistical analysis plan; only complete case analysis is reported, and results should be interpreted with caution.

^c^ Analyzed only for newborns who received postintervention surfactant (n = 75 in the intervention group; n = 78 in the control group).

^d^ Mann-Whitney *U* test indicates no significant difference in the overall distribution in days of mechanical ventilation between groups, although bootstrap CIs for the median demonstrate a difference in medians.

^e^ Defined as endotracheal ventilation; high-frequency oscillatory ventilation; continuous positive airway pressure; heated, humidified, high-flow nasal cannula oxygen; and low-flow nasal cannula oxygen.

^f^ Applicable only to newborns alive at 28 days of life.

^g^ Applicable only to newborns alive at 36 weeks after randomization.

^h^ Missing data were imputed using the mice package in R, version 3.14 (R Project for Statistical Computing), and results are pooled across 50 imputed data sets. Estimate and 95% CI are shown as the relative risk estimated from a log-binomial regression model, while the *P* value corresponds to a *z* test on the estimated coefficient.

^i^ Missing data were imputed using the mice package in R, version 3.14, and results are pooled across 50 imputed data sets. Estimate and 95% CI are shown for odds ratio calculated from a logistic regression model, while the test corresponds to a pooled *z* test for regression coefficients from the fitted models.

More newborns randomized to oropharyngeal surfactant were diagnosed with pneumothorax and were treated with needle aspiration or chest drain insertion (21 [16.6%] vs 8 [6.4%]; *P* = .04). All newborns diagnosed with pneumothorax were exposed to antenatal steroids. The incidence and duration of preterm premature rupture of membranes were similar in newborns diagnosed with pneumothorax in both groups. Among newborns diagnosed with pneumothorax, the mean GA and the proportion of newborns who received positive pressure ventilation and were intubated in the delivery room were similar in both groups.

There were no between-group differences in the secondary outcomes (Table 3), including rates of intubation in the delivery room (28 of 126 [22.2%] in the oropharyngeal group and 38 of 125 [30.4%] in the control group). Results of the sensitivity analysis of secondary end points did not differ substantially from results of the main analysis of secondary end points (eTable 9 in Supplement 2). Subgroup analyses were performed and are given in eAppendix 2 and eTables 10-12 in Supplement 2.

Among newborns who died before hospital discharge, all but 1 newborn met the primary outcome and were intubated within 120 hours of birth. This newborn, randomized to oropharyngeal surfactant, was first intubated on day-of-life 52 following a diagnosis of necrotizing enterocolitis. Rates of serious adverse events were similar between the groups (eAppendix 3 and eTables 13-16 in Supplement 2).

## Discussion

In this randomized clinical trial of 251 preterm newborns, surfactant administration into the oropharynx of newborns born before 29 weeks’ gestation immediately after birth in addition to CPAP, compared with CPAP alone, did not reduce the rates of intubation in the first 120 hours of life. Surfactant administration into the pharynx is a simple technique that avoids laryngoscopy and its associated short-term and long-term adverse effects. If the technique reduced the rate of ventilation, then it would hold the prospect of reducing adverse effects of intubation and mechanical ventilation. Our study did not find any benefit in using prophylactic oropharyngeal surfactant.

The rate of the primary outcome—intubation within 120 hours of life—in the control group (64.8%) was higher than our pretrial estimate (46%). We based our estimates on other trials^10,11,12,13^ of respiratory interventions in the delivery room. In contrast to other studies,^10,12,13^ we did not have a lower GA or birth weight cutoff, and preterm premature rupture of membranes was not an exclusion criterion. Furthermore, in our study, participants were randomized before birth, whereas in many delivery room studies, newborns only became eligible if they were breathing at 5 minutes after birth. Some participants in our study would have been unlikely to be enrolled in other studies (eg, those who weighed <500 g or had extensive resuscitation). In terms of eligibility and timing of randomization, our study was most similar to the Sustained Aeration of Infant Lungs (SAIL) trial^39^ and the Comparison of Respiratory Support After Delivery on Infants Born Before 28 Weeks’ Gestational Age (CORSAD) trial.^40^ More than half of the newborns in both groups in the SAIL trial were intubated in the delivery room,^39^ and more than one-third of infants in both groups were intubated in the delivery room in the CORSAD trial.^40^ In our study, among newborns of similar GA, rates of delivery room intubation were lower, regardless of group assignment.

Surfactant is a proven therapy for the prevention and treatment of RDS. We speculate that the lack of efficacy in our study was due to insufficient surfactant reaching the lungs. All newborns assigned to oropharyngeal surfactant received a dose of at least 100 mg/kg. Bohlin et al^30^ demonstrated that about half of the oropharyngeal surfactant administered to preterm rabbits reached the lungs. In our study, we were uncertain about how much surfactant was aspirated and speculate that it may have been highly variable among newborns.

There was an increased rate of pneumothorax in those assigned to oropharyngeal surfactant. Because type I error was not controlled among secondary outcomes, this difference may be attributable to the intervention, or it could have been a spurious finding.

### Strengths and Limitations

This study has strengths, including its multicenter design and timely completion. We found the small number of protocol violations reassuring, and the study was well-accepted by parents.

The main limitation of our study was that neither caregivers nor outcome assessors were blinded to group assignment. As there is no placebo that is safe for humans to aspirate, we considered using a sham procedure to attempt to mask the intervention. To attempt masking would have required a separate roster of study staff to attend out of hours. There was little prospect of having a separate team available at any participating center. This would have restricted enrollment to each center’s regular daytime working hours, and eligible newborns born outside regular daytime working hours could not be enrolled. In addition, we did not think that we could credibly blind caregivers, as the intervention was to be performed before cord clamping. We thought it unacceptable to limit enrollment to use a likely ineffective sham procedure. Ultimately, 60% of babies were enrolled outside of the centers’ regular daytime working hours; use of a likely ineffective sham procedure may have precluded the participation of many newborns.

## Conclusions

In this randomized clinical trial, administration of surfactant into the oropharynx immediately after birth in addition to CPAP compared with CPAP alone did not reduce the rates of intubation among newborns born before 29 weeks’ gestation in the first 120 hours of life. These results suggest that this technique should not be routinely used.

## Notes

Supplement 1.
Trial Protocol and Statistical Analysis Plan

Supplement 2. **eAppendix 1.** Sensitivity Analysis **eTable 1.** Primary Analysis of the Primary End Point **eTable 2.** Sensitivity Analysis 1 of the Primary End Point **eTable 3.** Sensitivity Analysis 2 of the Primary End Point **eTable 4.** Summary of Competing Risks for the Primary End Point **eFigure.** Cumulative Incidence Functions for Competing Risks for the Primary End Point **eTable 5.** Competing Risk Analyses for the Primary End Point **eTable 6.** Logistic Generalized Estimating Equation (GEE) Fitted to the Primary End Point, Adjusting the Estimated Treatment Effect for Potentially Relevant Covariates **eTable 7.** Sensitivity Analysis 5 for the Primary End Point **eTable 8.** Sensitivity Analysis 6 for the Primary End Point **eTable 9.** Sensitivity Analysis for Secondary End Points Originally Analyzed by Complete-Case Analysis **eAppendix 2.** Subgroup Analysis **eTable 10.** Relative Risk of Primary End Point, Intubation for Respiratory Failure Within 120 Hours of Birth, for Intervention vs Control Reported by Category of Gestational Age **eTable 11.** Relative Risk of Primary End Point for Intervention vs Control, by Center **eTable 12.** Log Binomial Regression Evaluating Whether Treatment Effect on the Primary End Point Differs by Gestational Age Subgroup **eAppendix 3.** Adverse Events **eTable 13.** Total Number of Adverse Events, Serious Adverse Events (SAEs), Severity, Relatedness to Treatment and Outcome of Adverse Events Occurring by Treatment Arm and in Total During the POPART Trial **eTable 14.** Subjects With Adverse Events, by Treatment Group **eTable 15.** Complete Listing of Serious Adverse Events (SAEs) **eTable 16.** Listing of Nonserious Adverse Events
eReference

Supplement 3.
Data Sharing Statement
