# Supplementary material for: Prophylactic Oropharyngeal Surfactant for Preterm Newborns at Birth: A Randomized Clinical Trial
Source: JAMA Pediatr. 2023 Dec 11;178(2):117–24. doi: 10.1001/jamapediatrics.2023.5082 (PMC10714282; doi:10.1001/jamapediatrics.2023.5082)
Supplement: Supplement 1. — Trial Protocol and Statistical Analysis Plan [file jamapediatr-e235082-s001.pdf]

POPART

1 STUDY TITLE

PROPHYLACTIC OROPHARYNGEAL SURFACTANT FOR PRETERM INFANTS: A RANDOMISED TRIAL (THE POPART TRIAL)

2 STUDY SPONSOR

University College Dublin
Belfield
Dublin 4
Ireland

3 APPLICATION DETAILS

3.1 Study title

PROPHYLACTIC OROPHARYNGEAL SURFACTANT FOR PRETERM INFANTS: A RANDOMISED TRIAL (THE POPART TRIAL)

A randomised trial of oropharyngeal surfactant versus no intervention at birth for infants born before 29 weeks of gestation to prevent endotracheal intubation for respiratory failure in the first 5 days of life

3.2 Reference numbers

Protocol identification (code or reference number): UCDCRC/16/003
EudraCT number: 2016-004198-41
Date and version number: 23-Nov-2020, Vers on 2.1

3.3 Applicant details

<u>Chief investigator/ Co-ordinating investigator</u>
Name/title:
Prof. Co m P.F. O Donne
Consultant Neonatologist, National Maternity Hospital
Professor, School of Medicine, University College Dublin
Department of Neonatology, National Maternity Hospital, Holles Street, Dublin 2

<u>Principal Investigators</u>
Name(s)/ titles:
Dr. Madeleine Murphy,
Research Fellow, National Maternity Hospital
Department of Neonatology, National Maternity Hospital, Holles Street, Dublin 2

<u>Funder :</u>
Name: Ch es Farmeceut c (Parma, Ita y).
Contact: Dr Besma Nash, PhD,
Med ca Sc ence L a son,
Ch es L m ted,
333 Styal Road, Manchester M22 5LG, United Kingdom

Sponsor
Name: Prof. Peter Doran
Contact detail s:
University College Dublin
Belfield
Dublin 4
Ireland
Peter.doran@ucd.ie

3.4 Sponsor & Chief Investigator Signatures

I, the undersigned, am responsible for the conduct of the trial and agree to the following:

- I understand and will conduct the trial according to the protocol, any approved protocol amendments, ICH GCP and all applicable regulatory authority requirements and national laws.
- I will not deviate from the protocol without prior written permission from the Sponsor and prior review and written approval from Independent Ethics Committee, except where necessary to prevent any immediate danger to the subject.
- I have read and understand fully the SmPC for Poractant alpha; and I am familiar with the Investigational Medicinal Product(s) (IMP) and its use according to this protocol.
- I have sufficient time to properly conduct and complete the trial within the agreed trial period, and I have available an adequate number of qualified staff and adequate facilities for the foreseen duration of the trial to conduct the trial properly and safely.

I will ensure that any staff at my site(s) who are involved in the trial conduct are adequately trained regarding the Investigational Medicinal Products, the protocol and their responsibilities. In the case of delegating any of my trial responsibilities I will provide the Sponsor with a Delegation of Activities certificate

	NAME	DATE
SPONSOR	*Prof Patrick Murray*	18 Jan 2021
CHIEF INVESTIGATOR	***Prof. Colm P.F. O'Donnell***	18-01-2021

PRINCIPAL INVESTIGATOR
PROTOCOL SIGNATURE PAGE

Protocol Title: **PROPHYLACTIC OROPHARYNGEAL SURFACTANT FOR PRETERM INFANTS: A RANDOMISED TRIAL (THE POPART TRIAL)**

Protocol Number: **UCDCRC/16/003 Protocol Version/**

Date: **Version 2.1, 23-Nov-2020**

Sponsor Name: **University College Dublin**

Declaration of Principal Investigator

I, the undersigned, am responsible for the conduct of the trial at this site and agree to the following:

- I understand and will conduct the trial according to the protocol, any approved protocol amendments, ICH GCP and all applicable regulatory authority requirements and national laws.
- I will not deviate from the protocol without prior written permission from the Sponsor and prior review and written approval from Independent Ethics Committee, except where necessary to prevent any immediate danger to the subject.
- I have read and understand fully the Summary of Product Characteristics for Poractant alpha; and I am familiar with the Investigational Medicinal Product(s) (IMP) and its use according to this protocol.
- I have sufficient time to properly conduct and complete the trial within the agreed trial period, and I have available an adequate number of qualified staff and adequate facilities for the foreseen duration of the trial to conduct the trial properly and safely.
- I will ensure that any staff at my site(s) who are involved in the trial conduct are adequately trained regarding the Investigational Medicinal Products, the protocol and their responsibilities. In the case of delegating any of my trial responsibilities I will provide the Sponsor with a Delegation of Activities certificate.

Principal Investigator Name: ______________________________

Principal Investigator Signature: ______________________________

Date (dd/mmm/yyyy): ______________________________

Site: ______________________________

5 TABLE OF CONTENTS

**6**	DOCUMENT HISTORY	5
**7**	SYNOPSIS	6
**8**	ABBREVIATIONS	7
**9**	INTRODUCTION	8
**10**	STUDY OBJECTIVE	9
**11**	TRIAL DESIGN	11
**12**	TREATMENT OF TRIAL SUBJECTS	20
**13**	SAFETY REPORTING	21
**14**	STATISTICS	26
**15**	DIRECT ACCESS TO SOURCE DATA/DOCUMENTS	29
**16**	DATA HANDLING AND RECORD KEEPING	29
**17**	RETENTION OF ESSENTIAL DOCUMENTS	30
**18**	QUALITY CONTROL AND QUALITY ASSURANCE PROCEDURES	30
**19**	AUDITS AND INSPECTIONS	31
**20**	ETHICS	31
**21**	FINANCING AND INSURANCE/INDEMNITY	32
**22**	CLINICAL STUDY REPORT AND PUBLICATION POLICY	33
**23**	REFERENCES	33

6 DOCUMENT HISTORY

Document	Date of Issue	Summary of Change
Version 1.0 - Original protocol	28-Nov-2016	Not applicable
Vers on 1.1	09-Jan-2017	The protoco has been rev sed fo ow ng queries/recommendations from the PRA on 21st December 2016:<br>• Sect on 10. 3 Pr mary Endpo nt th s has been changed throughout the protoco to "the nc dence of endotrachea ntubat on for resp ratory fa ure w th n 12 hours of b rth" as per PRA recommendation<br>• Section 10 3 Secondary Endpoints provides further clarity on method of measurement for outcomes<br>• Section 11 Study Design Further clarity provided on the eve of care on each of the arms of the study.<br>• Section 11 1 Timing of administration of surfactant defined for the ntervent on arm.<br>• Section 11 2 3 Exclusion Criteria (and also Synopsis) of the protoco rev sed to prov de further deta on cr ter a: majore congenital abnormalities<br>• Section 11 3 Schedule of Events this has been adapted to separate the Emergency/non routine procedures<br>• Section 11 3 1 Description of Study Assessments further detail added on the assessment of Rectal Temperature and also recording of concomitant medications<br>• Section 11 3 4 Baseline Assessments further information on recording of assessments provided<br>• Section 11 3 5 Subsequent Study Visits 'the assessment of incidence' of intubation at 120 hours is the goal' is added<br>• Section 12 5 Assessment of Compliance further clarity provided on how compliance with study medication will be assessed.
Vers on 1.2	06-Nov-2018	Non substantial Protocol Amendment<br>• Protocol Signature page for Principal nvestigator added<br>The following sections of the protocol were revised to provide further c ar ty spec f ca y for the competent author ty n Sweden the MPA<br>• Section 12 2 Formulation packaging and handling Omission of requ rement for separate abe ng of IMP for Swed sh sites Curosurf will be the same as routinely used in the N CU for Swedish sites<br>• Sect on 14 Stat st cs Further nformat on prov ded on the ana ys s sets, cr ter a for term nat on of the tr a and the eve of statistical significance
Vers on 2.0	19-Jul-2019	Protocol Amendment<br>• Section 13 Safety Reporting Updates made to provide further c ar ty on the process for report ng of d fferent medical and safety events occurring in participants<br>• Section 14 5 Efficacy Analysis Updates made to statistical analysis plan<br>• Section 16 1 Data collection source documents and case report forms (CRF) Added deta on process for data collection in cases where patient is transferred to a different hosp ta
Vers on 2.1	23-Nov-2020	Non substantial Protocol Amendment issued<br>• Section 11 4 Definition of end of trial Further clarity provided on activities related to end of trial and beginning of analysis<br>As study has begun close out activities at certain sites Protocol signature page may not be signed by all P s in sites if country local End of Trial has already been submitted and/ors site has been closed out

7 SYNOPSIS

Title of study	POPART tr a
Name of sponsor/company	University College Dublin
Phase of development	Phase 3
Objectives	To determine whether, among infants born before 29 weeks of gestation, does oropharyngeal surfactant at birth compared to no intervention reduce the rate of endotracheal intubation for respiratory failure within 120 hours of birth
Tr a des gn	Randomised, parallel group, controlled trial
Key inclusion criteria	• Infants born before 29 weeks of gestation<br>• Initiation of intensive care
Key exc us on cr ter a	• Major congenital anomalies including neural tube defects, major structural cardiac anomalies (excluding PDA/ASD/VSD), abdominal wall defects and congenital diaphragmatic hernia and major dysmorphic features with an abnormal karyotype e.g. T21, T13, T18<br>• Non-initiation of intensive care
Number of subjects	250
Test product, dose and mode of administration	Curosurf (Ch es Farmeceut c , Parma, Ita y) 120mg or 240mg given by injection into the oropharynx
Duration of treatment	Once off administration (c. 10 seconds)
Statistical methods	Comparison of the primary outcome (intubation for respiratory failure within 120 hours of birth) and each dichotomous secondary outcome between oropharyngeal surfactant at birth and no intervention, using a two-sided two proportion Z test.<br>Comparison of continuous secondary outcomes between oropharyngeal surfactant at birth and no intervention, using independent samples t-tests.<br>Regression analysis will be carried out to determine the sensitivity of the treatment effect to relevant covariates (such as gestational age)
Samp e s ze	A sample size of 250 is required detect a difference of 18% in the rate of intubation between infants receiving oropharyngeal surfactant at birth and no intervention with a statistical power of 80%, at a significance level of 5%. Th s ca cu at on assumes an ant c pated oss-to-follow-up of 10% of recruited infants.

8 ABBREVIATIONS

AE	Adverse event
AR	Adverse react on
BPD	Bronchopu monary dysp as a
CA	Competent authority
CGA	Corrected gestat ona age
CI	Chief investigator/Co-ordinating investigator
CLD	Chronic lung disease of prematurity
CPAP	Cont nuous pos t ve a rway pressure
CRA	C n ca research assoc ate
CRF	Case report form
CRO	Contract research organ sat on
CT	Clinical trial
CTA	Clinical trial authorisation
CXR	Chest x-ray
DR	Delivery room
DSMB	Data Safety Monitoring Board
ECG	Electrocardiogram
ETT	Endotrachea Tube
EU	European Un on
e-CRF	Electronic case report form
GCP	Good C n ca Pract ce
GP	General Practitioner
IB	Investigators brochure
ICF	Informed consent form
ICH	International Conference on Harmonisation
IEC	Independent Ethics Committee
IMP	Investigational medicinal products
IMPD	Investigational medicinal product dossier
IVH	Intraventricular haemorrhage
HPRA	Health Products Regulatory Authority
HSE	Hea th Serv ce Execut ve
NICU	Neonatal intensive care unit
PI	Principal investigator
PIL	Patient/subject information leaflet
REC	Research eth cs comm ttee
RDS	Resp ratory D stress Syndrome
ROI	Republic of Ireland
SAE	Ser ous adverse event
SAR	Ser ous adverse react on
SmPC	Summary of product characteristics
SOP	Standard operating procedure
SUSAR	Suspected unexpected ser ous adverse react on

9 INTRODUCTION

9.1 Background information

All newly-born infants have fluid-filled lungs. Within a short time after birth, they must stop producing this liquid, clear it from their lungs and replace it with air. Respiratory distress syndrome (RDS) is a lung condition characterised by difficulty in recruiting and maintaining an adequate volume of gas in the lungs. It manifests with increasing signs of respiratory distress (tachypnoea; grunting; subcostal, intercostal and sternal recession) and evidence of respiratory failure (supplemental oxygen requirement; acidosis and hypercarbia on blood gas analysis) in newborns at or shortly after birth. The diagnosis is made in infants who have typical chest radiograph changes and do not have another explanation (e.g. perinatal sepsis) for their respiratory failure. The risk of RDS is inversely related to gestational age, such that it is rarely seen in term infants and seen in the majority of infants born at 24 weeks.

Infants with RDS have structural and functional immaturity of their lungs. Extremely premature infants have poorly developed alveoli, relatively weak respiratory musculature and pliable chest walls. They also have a relative lack of surfactant, an endogenously produced substance that enables alveoli to expand more easily to recruit and maintain gas within the lung. Exogenous surfactant is frequently used to treat newborns with RDS. Surfactant is instilled directly into the trachea though an endotracheal tube (ETT). The ETT is introduced under direct vision using a laryngoscope, a metal instrument with a light at the end designed to lift the tongue out of the way so that the vocal cords can be seen. When it was introduced in the 1990s, exogenous surfactant led to a reduction in mortality among premature infants with RDS. In more recent years, however, managing premature newborns initially with continuous positive airway pressure (CPAP) and reserving intubation, mechanical ventilation and surfactant for those infants with worsening respiratory failure despite CPAP, has yielded better results than intubating all infants for surfactant administration.(1) However, nearly half of infants initially managed with CPAP are ultimately intubated for surfactant and ventilation. (2)

Intubation is a procedure that is difficult to learn and is associated with adverse effects, both short (e.g. pain, hypoxaemia, bradycardia, oropharyngeal trauma) and longer-term (e.g. chronic lung disease, subglottic stenosis). This has led many clinicians to investigate alternative methods of surfactant delivery. Giving nebulised surfactant to spontaneously breathing infants has met with limited success. Progress has been slow due to the technical difficulties encountered in aerosolising such large molecules, the expense of the equipment needed to do so and the expense of the large amount of surfactant needed to form an aerosol. Interest has thus largely focussed on less-invasive methods of surfactant administration. The “minimally invasive” techniques have involved introducing either a feeding tube or vascular catheter into the trachea of a spontaneously breathing infant under direct vision with a laryngoscope.(3) These techniques may reduce the need for mechanical ventilation among preterm infants. However, they appear more difficult than intubation and the many short term adverse effects of intubation that are due to laryngoscopy are not avoided. Direct administration of surfactant into the pharynx of human infants has been described in randomised studies and prospective cohort studies.(4, 5) It is apparently effective and is an easier technique to perform than endotracheal intubation or passing a feeding tube or vascular catheter into the trachea.

We will perform a study to establish whether giving preterm infants surfactant into their oropharynx at birth reduces their need for subsequent intubation in the first 5 days of life.

The population to be studied include infants born at less than 29 weeks by best obstetric estimate where the treating physician intends to offer intensive care.

9.2 Rationale for the study

Poractant alpha (Curosurf, Chiesi Farmeceutici, Parma, Italy) is a natural surfactant prepared from porcine lungs. Large randomised controlled trials in the 1980s – 1990s(6-8) demonstrated that poractant alpha given by endotracheal tube (ETT) reduced mortality(4, 9) and air leak among premature infants who were intubated for respiratory failure due to the respiratory distress syndrome (RDS). This led to the widespread practice of intubating all extremely preterm infants for surfactant and ventilation

(“prophylactic surfactant”)(7). Poractant alpha is approved for endotracheal use for prevention and/or treatment of RDS in premature infants.

Concerns were raised in the 1980s – before the introduction of surfactant into clinical practice – that premature infants who were intubated for respiratory support had worse respiratory outcomes than infants who were managed with the non-invasive respiratory support nasal continuous positive airway pressure (NCPAP)(10). These concerns persisted through the 1990s – 2000s, i.e. after the widespread introduction of surfactant. Randomised trials performed in the 2000s demonstrated that starting infants on NCPAP and reserving intubation and surfactant for infants who deteriorate, increases the rate of survival without chronic oxygen dependence (chronic lung disease of prematurity)(11). About half of premature infants who start on NCPAP for RDS are ultimately intubated and given surfactant(2).

Our study will examine oropharyngeal administration of surfactant given as prophylaxis for RDS, to infants at risk of RDS. The more prematurely an infant is born, the higher their risk is of being intubated for respiratory support in the first 120 hours of life (our primary outcome). As infants born less than 29 weeks are most at risk of RDS, these infants will be included in our study.

Side effects seen following the administration of Curosurf include bradycardia, hypotension, endotracheal tube blockage and oxygen desaturation.

It is licensed for endotracheal use for the prevention and treatment of RDS in preterm infants. It is currently not licensed for oropharyngeal administration, and therefore this study will examine the off-label use of a licensed product.

The recommended dosing regimen for prophylaxis and rescue treatment of RDS is 100-200mg/kg given endotracheally.

10 STUDY OBJECTIVE

To determine whether administering oropharyngeal surfactant to premature infants at birth reduce the rate of intubation for respiratory failure in the first 5 days of life.

Hypothesis: Oropharyngeal surfactant reduces the rate of endotracheal intubation in preterm infants in the first 5 days of life.

Research quest on:
(P) Among infants born at less than 29 weeks of gestation,
(I) does the administration of surfactant into the pharynx by mouth at birth
(C) compared to no intervention
(O) reduce the rate of endotracheal intubation for respiratory failure
(T) in the first 120 hours of life?

The National Maternity Hospital is a stand-alone university maternity hospital with a tertiary NICU to which >150 infants <1500g are admitted annually. Approximately 60 babies <29 weeks gestation are admitted annually. We have a track record of initiating, conducting, completing, presenting and publishing investigator-led randomised controlled trials, including trials that compared delivery room interventions,(12-14) trials that had respiratory failure in the NICU as the primary outcome(15) and trials that compared investigational medicinal products(16) in preterm infants. Though the enrolment rates to our studies amongst eligible infants are consistently excellent (> 80%), we believe it will be necessary to enrol infants at multiple sites in order to enrol our planned target sample of 250 infants in a timely fashion. We have a track record enlisting the help of collaborators nationally(16) and internationally(14, 17) to perform our studies. We believe that with their help, we can enrol these infants in 3 years.

10.1 Primary objective

Our primary objective is to investigate the efficacy of prophylactic oropharyngeal surfactant for reducing the rate of endotracheal intubation, compared to no intervention in infants at risk of RDS.

10.2 Secondary objective

To investigate the efficacy of prophylactic oropharyngeal surfactant compared to no intervention for preventing some possible complications in premature infants at risk of RDS, we will also record a number of variables relating to prematurity, from enrolment into the trial until discharge home from hospital.

10.3 Primary and secondary/exploratory endpoints/outcome measures

The primary outcome is the incidence of endotracheal intubation for respiratory failure within 120 hours of birth.

<u>Primary outcome</u>

The incidence of endotracheal intubation for respiratory failure within 120 hours of birth. Enrolled infants will be intubated for persistent apnoea and/or bradycardia in the DR, or for respiratory failure in the NICU defined as ≥ 2 of:

- Clinical signs – worsening tachypnoea; grunting; subcostal, intercostal and/or sternal recession
- Acidosis – pH < 7.2 on 2 blood gases (arterial or capillary) ≥ 30 minutes apart
- O_2 – FiO_2 > 0.4 to keep SpO_2 ≥ 90% for > 30 minutes
- PCO_2 > 9.0 kPa on 2 blood gases (arterial or capillary) ≥ 30 minutes apart
- Apnoea – recurrent apnoea treated with mask ventilation

The frequency of blood gas monitoring is based on the clinical decision of the treating physician, as per routine practice.

Enrolled infants will only be intubated if they reach the pre-determined criteria for respiratory failure. After giving endotracheal surfactant for the treatment of RDS, attending clinicians may attempt to extubate the babies immediately ("INSURE") or they may elect to ventilate the babies for a longer period. This is at their discretion.

<u>Secondary outcomes</u>

- Intubation in the delivery room (DR)
- Number of attempts taken to successfully intubate in the DR
- Chest compress ons n the DR
- Adrenaline administration in the DR
- Rectal temperature on admission to the NICU
- NICU intubation
- Surfactant use before death or hospital discharge
    - Number of doses, including total dose
    - Intra-tracheal surfactant received post-intervention
    - Doses of post-intervention surfactant
- Resp ratory d stress syndrome
    - Clinical evidence of respiratory distress with radiological evidence (ground glass appearance on CXR)
- Incidence of pneumothorax
    - Incidence of pneumothorax on CXR
- Incidence of pulmonary haemorrhage
    - Clinical evidence of pulmonary haemorrhage
- Mechanical ventilation
- Days of mechanical ventilation
- Use of postnatal corticosteroids for ventilator dependence
- Days of duration of respiratory support (endotracheal ventilation, high-frequency oscillatory ventilation, CPAP, heated humidified high-flow nasal cannula O_2, low flow nasal cannula O_2)
- Bronchopulmonary dysplasia (BPD) – supplemental O_2 at 28 days of life
- Chronic lung disease of prematurity (CLD) – need for supplemental O_2 at 36 weeks corrected gestational age (CGA) determined by physiological oxygen reduction test
- Medical treatment for a patent ductus arteriosus
    - Administration of ibuprofen or paracetemol for PDA

- Surgical treatment for a patent ductus arteriosus
- Proven necrotising enterocolitis (≥ Bell's stage 2)
- Incidence of Intraventricular haemorrhage (IVH) (any and severe: IVH grade ≥ 3)
    - Evidence on surveillance cranial ultrasounds performed regularly in NICU as standard of care
- Incidence of cystic periventricular leukomalacia
    - Evidence on surveillance cranial ultrasounds performed regularly in NICU as standard of care
- Retinopathy of prematurity treated with laser photocoagulation or intravitreal injections
    - Evidence on surveillance ophthalmology review performed regularly in NICU as standard of care
- Death before hospital discharge
- Survival without BPD at hospital discharge
- Survival without CLD at hospital discharge
- Duration of hospitalisation
- Use of home oxygen therapy
    - Discharged home on oxygen therapy

These are clearly defined outcome measures and will be recorded during the infants hospital stay prior to discharge home from hospital. These outcome measures include complications of prematurity that can occur at any stage from birth until discharge home and thus specific timeframes for measurement is not applicable in all cases.

11 TRIAL DESIGN

11.1 General considerations

This will be an international, multicentre, randomised parallel group control trial, however initially will be single centre. It will be conducted in accordance with good clinical practice guidelines and all applicable regulatory rules under the guiding principles of the Declaration of Helsinki.
Infants born less than 29 weeks gestational age will be included if the treating physician plans to offer intensive care. Infants will be excluded if infants have major congenital anomalies and if the treating physician does not plan to offer intensive care. Written informed consent will be obtained before delivery.

Figure 1: Study Schema

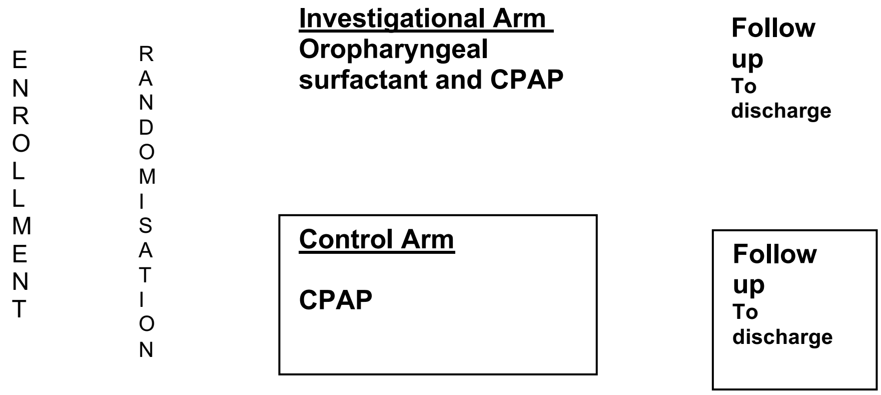

Prior to delivery a member of the research team or other senior doctor will approach parent(s)/guardian(s) of eligible infants to inform them about the study.
Randomisation will occur at delivery. Infants will then be monitored for the duration of their hospitalisation, looking at a large number of secondary outcomes. Subjects' participation will be from time of birth until discharge from hospital.

Infants in the treatment arm will receive oropharyngeal surfactant immediately after birth and then be stabilised on CPAP. Infants in the control arm will be stabilised on CPAP immediately after delivery. Infants will then receive standard care, regardless of their group assignment. Enrolled infants will be intubated for persistent apnoea and/or bradycardia in the DR, or for respiratory failure in the NICU as specified in the protocol. Should infants in either group meet the criteria for respiratory failure following initial randomisation, they will be intubated and surfactant will be administered for treatment of RDS, irrespective of group assignment (i.e. whether or not they have previously received oropharyngeal surfactant). Further surfactant administration and all other aspects of neonatal intensive care will be at the discretion of the treating physicians.

All babies in both groups will be closely watched to see if they need extra treatment for their RDS at any stage, including surfactant given endotracheally. The babies will be treated equally and given whatever treatment is necessary, whether or not they have already been given surfactant into the oropharynx.

INTERVENTION

Care givers will not be masked to group assignment.

OROPHARYNGEAL SURFACTANT GROUP

Infants randomised to oropharyngeal surfactant will receive a dose of surfactant (Curosurf, Chiesi Farmeceutici, Parma, Italy) (18) immediately after birth, ideally before the cord is clamped e.g. 60 seconds. If it is given after the cord is clamped it will be given once the infant is placed on the resuscitaire. It will be given within 5 minutes of birth in all cases.

The surfactant will be warmed prior to being drawn up in a sterile syringe as per manufacturer's recommendation. This will be done by opening the mouth gently and administering the surfactant as a single bolus into the oropharynx using a syringe without a needle attached. This will be done as soon as possible after delivery, ideally before the umbilical cord has been clamped.

The dosing recommendations for initial treatment with Curosurf when given by endotracheal tube are:

- 200mg/kg for estab shed RDS
- 100 – 200mg/kg for prophylaxis

One or two further doses of 100mg/kg Curosurf may be given to infants who have persistent respiratory distress despite treatment with surfactant (maximum recommended dose 400mg/kg).

The timing or dosage of ET surfactant will not be affected by initial oropharyngeal surfactant. If an infant is felt to need ET surfactant following initial oropharyngeal administration, then they will receive the standard initial dose of 200mg/kg via ETT.

Infants in our study will not be weighed prior to enrolment. The 50th centile for birth weight (BW) for boys and girls according to gestational age (GA) is shown below

GA (weeks)	G r s BW (kg)	Boys BW (kg)
23	0.550	0.600
24	0.650	0.700
25	0.775	0.800
26	0.850	0.900
27	0.975	1.050
28	1.100	1.150

In our study, infants < 26 weeks will receive a full 120mg vial of Curosurf. We estimate that this will provide dosing in the therapeutic range as indicated below:

GA (weeks)	G r s BW (kg)	Dose (mg/kg)	Boys BW (kg)	Dose (mg/kg)
23	0.550	218	0.600	200
24	0.650	185	0.700	171

25	0.775	155	0.800	150

In our study, infants 26–28 weeks will receive a full 240mg vial of Curosurf. We estimate that this will provide dosing in the therapeutic range as indicated below:

GA (weeks)	G r s BW (kg)	Dose (mg/kg)	Boys BW (kg)	Dose (mg/kg)
26	0.850	282	0.900	267
27	0.975	246	1.050	229
28	1.100	218	1.150	209

CONTROL GROUP

Infants randomised to the control group will not have anything injected into their oropharynx and will be stabilised on CPAP in the delivery room (DR) as per routine practice.

11.2 Selection of study population

11.2.1 Overall description of trial subjects

Trial subjects will be premature babies at risk of respiratory distress syndrome.

11.2.2 Inclusion criteria

Infants will be eligible for enrolment if

- they are born at a participating centres with a gestational age at birth < 29 weeks (i.e. up to 28^{+6} days gestation) by best obstetric estimate; and
- the treating doctors plan to offer them intensive care

Infants of multiple gestation and of either sex are eligible to be enrolled.

To be eligible for inclusion, each subject must meet each of the following criteria at Screening (Visit 1) and must continue to fulfil these criteria at Baseline (Visit 2).

- Subjects' parents(s)/guardian(s) will be approached for written informed consent for enrolment of the infant in the study prior to delivery.
- Subjects will be preterm babies less than 29 weeks gestation at baseline, of either gender.
- Subjects will be at risk of respiratory distress syndrome.
- Subjects' clinicians must plan to offer them intensive care

11.2.3 Exc us on cr ter a

Subjects are excluded from the study if any of the following criteria are met at Screening (Visit 1) or at Base ne (V s t 2):

- Infants with major congenital anomalies including neural tube defects, major structural cardiac anoma ies (exc uding PDA/ASD/VSD), abdomina wa l defects and congenita diaphragmatic hernia and major dysmorphic features with an abnormal karyotype e.g. T21, T13, T18.
- The treating clinician does not intend to offer the infant intensive care.
- Written informed consent has not been obtained prior to delivery or subjects' parent(s)/guardian(s) withdraws consent following enrolment.

11.3 Study assessments and procedures

Figure 2: Schedule of Events

**Procedures**	**Visit 1**<br>Screening	**Visit 2**<br>Baseline<br>Day 0- Day of Birth	**Visit 3**<br>120 hours after birth	**Visit 4**<br>Day 28 of life	**Visit 5**<br>CGA 36 weeks	**Visit 6**<br>End of Study V s t<br>D scharge home
Inclusion/Exclusion Criteria	X	x				
Informed consent	X	x				
Demographics		x				
Randomisation		x				
Dispensing of study medications		x				
V ta S gns		x				
Phys ca Exam nat on		x				x
Medication comp ance check		x				
Rectal Temperature		x				
Adverse event assessments		x	x	x	x	x
Concomitant medications		x	x	x	x	x
Intubation for respiratory failure			x			
Assessment of Bronchopu monary dysplasia				x		
Assessment of Chron c ung d sease of prematurity					x	
Assessment of Other secondary outcomes						x
**Emergency/non-routine procedures:**						
Assessment of the incidence of chest compressions		x				
Adrenaline Administration		x				

Prior to delivery a member of the research team or other senior doctor will approach parent(s)/guardian(s) of eligible infants to inform them about the study. The team member will explain the purpose and nature of the study and provide written information for the parent(s)/guardian(s) to keep. Parent(s)/guardian(s) will be offered the opportunity to have another family member or support person present while the study is explained. If English is not their first language, they will also be offered the opportunity to have an interpreter present while the study is explained.

Written consent for enrolment of the infant in the study will then be sought. Parents will be informed that they may withdraw their child from the study at any time should they so wish; and that a decision not to consent to their infants participation in the study or to withdraw their infant from the study once enrolled will not affect their infant's access to the best available treatment and care at the birth hospital or other hospital to which they might be referred. Consent forms will be kept securely and a copy will be provided to the parents for their records.

Infants will be randomised at delivery as outlined separately. Infants randomised to oropharyngeal surfactant will receive a dose of surfactant. Infants randomised to the control group will not have anything injected into their oropharynx and will be stabilised on CPAP in the DR as per routine practice.

Our primary outcome is the incidence of endotracheal intubation for respiratory failure within 120 hours of birth. Other secondary outcomes have predefined outcome measures and will be recorded on the individual CRF during the course of hospitalisation.

11.3.1 Descr pt on of Study Assessments

The following study assessments and procedures will be conducted as part of the clinical trial:

<u>Demographics</u>
The date of birth, gender and race of the infant will be recorded. Gestation, multiplicity, birth weight and resuscitation at delivery, including Apgar scores, will be documented.
Maternal age will be recorded. We will also record administration of antenatal steroids, preterm premature rupture of membranes (evidence of and duration of), intra-partum antibiotic use, the mode of delivery and indication for mode of delivery.

<u>Phys ca Exam nat on</u>
The complete physical examination of the infant will include the evaluation of the cardiovascular, dermatological, musculoskeletal, respiratory, gastrointestinal, neurological systems. Weight and rectal temperature will be recorded.

<u>V ta S gns</u>
Vital signs will be recorded for all subjects and will include: blood pressure (BP), temperature (C), pulse, and respiratory rate. Vital signs will be obtained at Baseline.

<u>Concomitant Medications</u>
Medications to be documented on the CRF include

- Postnatal corticosteroids for ventilator dependence
- Medical treatment for a patent ductus arteriosus
- Medical management of Necrotising enterocilitis (≥ Bell's stage 2)
- Intravitreal injection for retinopathy of prematurity

No medications are restricted in the treatment of the infants as a result of participating in this trial.

<u>Chest Compress ons</u>
Chest compress ons de vered n DR

<u>Rectal Temperature</u>
Rectal temperature on admission to NICU. Recording rectal temperature in newborns provides the most accurate temperature recording in comparison with other methods and is performed routinely in NICUs.

<u>Adrenaline Administration</u>
Adrenaline administration by intravenous or endotracheal route in DR

<u>Intubation for Respiratory Failure</u>
Respiratory failure in the NICU defined as ≥2 of:
Clinical signs – worsening tachypnoea; grunting; subcostal, intercostal and/or sternal recession
Acidosis – pH <7.2 on 2 blood gases (arterial or capillary) ≥ 30 minutes apart
O_2 – FiO_2 > 0.4 to keep S_pO_2 ≥ 90% for > 30 minutes
PCO_2 > 9.0 kPa on 2 blood gases (arterial or capillary) ≥ 30 minnutes apart
Apnoea – recurrent apnoea treated with mask ventilation

<u>Assessment of Bronchopu monary Dysp as a</u>
supplemental O_2 therapy at 28 days of life

Assessment of Chron c Lung D sease Prematur ty

Need for supplemental O_2 at 36 weeks corrected gestational age (CGA) determined by physiological oxygen reductiontest

11.3.2 Endpo nts assessments

Eff cacy Assessment

The primary outcome – whether the infant has been intubated for respiratory failure – will be determined at 120 hours of life.

The following secondary outcomes will be determined at the times indicated:

- Intubation in the delivery room (DR)
- Number of attempts taken to successfully intubate in the DR
- Chest compress ons n the DR
- Adrenaline administration in the DR
- Rectal temperature on admission to the NICU
- Bronchopulmonary dysplasia (BPD) – supplemental O_2 at 28 days of life
- Chronic lung disease of prematurity (CLD) – need for supplemental O_2 at 36 weeks corrected gestational age (CGA) determined by physiological oxygen reduction test
- Death before hospital discharge

The following secondary outcomes will be measured during the infant's hospitalisation and determined at d scharge:

- NICU intubation
- Surfactant use
    - Number of doses, including total dose
    - Intra-tracheal surfactant received post-intervention
    - Doses of post-intervention surfactant
- Incidence of pneumothorax
- Incidence of pulmonary haemorrhage
- Mechanical ventilation
- Days of mechanical ventilation
- Use of postnatal corticosteroids for ventilator dependence
- Days of duration of respiratory support (endotracheal ventilation, high-frequency oscillatory ventilation, CPAP, heated humidified high-flow nasal cannula O_2, low flow nasal cannula O_2)
- Medical treatment for a patent ductus arteriosus
- Surgical treatment for a patent ductus arteriosus
- Proven necrotising enterocolitis (≥ Bell's stage 2)
- Incidence of Intraventricular haemorrhage (IVH) (any and severe: IVH grade ≥ 3)
- Incidence of cystic periventricular leukomalacia
- Retinopathy of prematurity treated with laser photocoagulation or intravitreal injections
- Survival without BPD at hospital discharge
- Survival without CLD at hospital discharge
- Duration of hospitalisation
- Use of home oxygen therapy

Safety Assessment

The following safety evaluations will be performed during the study: adverse event monitoring. Surfactant has an excellent safety profile for use. Previously reported adverse effects include bradycardia and desaturation.

We will also record the number of intubation attempts in DR post oropharyngeal surfactant use.

11.3.3 Screen ng procedure

Names of potential participants' parents(s)/guardians(s) will be obtained from the obstetric team, antenatal wards and clinics. They will be approached by a member of the research team or other senior doctor (neonatal consultant or registrar) to inform them of the study. We intend to recruit where possible, however it is likely the majority will be recruited when in-patients in the hospital e.g. antenatal wards or delivery ward. An information sheet will be provided to the parents to keep and the details of the study will be explained to them. They will then be invited to participate in the study.

Once informed consent is obtained, should preterm delivery prior to 29 weeks gestation ensue, then randomisation will occur at the time of delivery. There is no maximum duration between screening and randomisation, as long as delivery is prior to 29 weeks gestation. Informed consent is the only procedure that must be completed before the study begins.

During the screening period subjects will be evaluated for eligibility. Assessment of inclusion/exclusion criteria will be again assessed at the time of randomisation for the subject to continue in the study.

Informed consent will be obtained prior to enrolment in the trial.

11.3.4 Base ne assessments

The following pre-treatment Baseline assessments/procedures will be performed prior to randomisation:

- confirmation of eligibility (review inclusion/exclusion criteria)
- informed consent

Following randomisation and obtaining consent, the following assessments/procedures will be done at baseline, (the day of birth):

- dispensing of study medication
- collection of vital signs, concomitant medications and demographics information
- phys ca exam nat on
- rectal temperature
- chest compress ons
- administration of adrenaline
- adverse events assessment

The recording of the incidence of chest compressions and adrenaline administration is the aim as these are not necessarily routine procedures.

11.3.5 Subsequent study v s ts and procedures

The intervention in this study is a once-off administration of surfactant at birth. After this intervention, all other cares will be the same between infants in both arms.
Assessment of the incidence of intubation for respiratory failure is the goal and will be conducted at 120 hours post birth (visit 3).
Assessment of bronchopulmonary dysplasia will be at day 28 of life and assessment of chronic lung disease prematurity will be at CGA 36 weeks.

During hospitalisation, at all visits after and including baseline visit, the following assessments/procedures w occur:

- assess eff cacy outcome measures
- assess safety (adverse event mon tor ng)
- d spense study med cat ons
- record concomitant medications
- assess comp ance w th study med cat ons

11.3.6 Method of assigning Subjects to treatment groups

Infants will be randomised to receive oropharyngeal surfactant or CPAP (which is the standard of care) in a 1:1 ratio using variable block randomisation, with block sizes of 4, 6 and 8. Randomisation will be stratified by participating centre. The more prematurely an infant is born, the higher their risk is of being

intubated for respiratory support in the first 120 hours of life (primary outcome). It is important to ensure balance in the risk of reaching the primary outcome between the two treatment arms at study entry; thus we will also stratify the randomisation by gestational age (<26 weeks and 26-28 weeks inclusive).

Infants of multiple gestations will be randomised as individuals.

A computer-generated randomisation schedule will be prepared by an independent statistician who will not be involved with subsequent data analysis or interpretation. The schedule will be stored securely on a password protected computer. The randomization list will be numbered using sequential 6-digit randomization codes (e.g. NMH001, NMH001,…). Each group assignment – "OROPHARYNGEAL SURFACTANT" or "CONTROL" – will be written on a card that will be labelled with its corresponding 6-digit randomisation code (e.g. NMH001). Each card will be folded in half and placed in an opaque sealed envelope that will be labelled with the same 6-digit randomisation code. The envelopes will then be placed in sequential order (e.g. NMH001, NMH002, NMH003…) in separate boxes for the two gestational age strata for each centre. At each centre, the boxes containing the envelopes will be stored securely in the neonatal intensive care unit (NICU), as the clinical team will need to have access to the randomisation envelopes. An envelope from the appropriate box (<26 weeks or 26–28 weeks) will be will be opened immediately before birth.

Blinding

This is an open-label study. The study will not be blinded to investigator, subjects, or medical or nursing staff. We are not using a placebo, and in the event of the baby being randomised to the 'CONTROL' arm, then they will be commenced on CPAP immediately after birth.
The trial statistician will be blinded for data analysis, and will be kept unaware of treatment group assignments. The randomization schedule will be drawn up by an independent statistician, to facilitate this.

11.4 Definition of end-of-trial

The end of the trial will be when all infants have been recruited and the last baby has been discharged from hospital, in order to ensure all secondary outcomes have been recorded. On occasion, surviving premature infants have a surgical intervention (e.g. tracheostomy, bowel resection) that renders them dependent on technology (e.g. positive pressure respiratory support, parenteral nutrition). These infants may remain as hospital in-patients for months or years beyond their expected date of delivery (EDD). It is not possible to identify these infants at enrolment. If one such infant is enrolled in the study, waiting to collect their discharge-based secondary outcome data (date of discharge, duration of hospitalisation, receipt of home oxygen) would indefinitely defer the analysis of the primary outcome and other secondary outcome data for all participants. After enrolment is complete, it will be determined whether any infant who remains in hospital one month beyond their EDD remains in receipt of supplemental oxygen and their primary and other secondary outcome data (up to Section I of the Case Record Form) will be locked for analysis at that point. These infants will be followed to ascertain their date of discharge and duration of hospitalisation (Section J). The participation of infants who remain as in-patients 6 months after their EDD will be censored, and their duration of hospitalisation will be recorded at that point.

The Sponsors and/or the trial steering committee, however, have the right at any time to terminate the study for clinical or administrative reasons.
The end of the study will be reported to the REC and Regulatory Authority within 90 days, or 15 days if the study is terminated prematurely. The investigators will inform subjects and ensure that the appropriate follow-up is arranged for all involved.
A summary report of the study will be provided to the REC and Regulatory Authority within 1 year of the end of the study and within 6 months for paediatric studies. This is a legal requirement.

The end-of-trial is the date of the last visit/telephone follow-up/ home visit of the last subject. The end of study visit form should include:

- assessment of endpo nts/outcome measures

- assessments of safety including adverse event collection
- assessment of compliance with study treatment(s)
- recording of concomitant medications

11.4.1 Premature termination of the study

The study can be prematurely terminated on the advice of the Data Safety Monitoring Board if safety concerns are detected at any time during the study.

The study may also be prematurely terminated in case of overwhelming evidence of efficacy or futility, defined as highly statistically significant difference (i.e. $P < 0.001$) in the primary outcome or a highly statistically significant difference in the important secondary outcome death before hospital discharge, from interim analysis. Interim analysis for efficacy or futility will be carried out after approximately 50% of participants have completed the study (i.e. after the completion of 126 patients) using the Haybittle- Peto stopping boundary.

11.5 Discontinuation/withdrawal of subjects from study protocol

Subjects have the right to voluntarily discontinue study treatment or withdraw from the study at any time for any reason without any consequences. Subjects will be withdrawn from the study for any of the following reasons:

- withdrawal of consent by the subjects' parent(s)/guardian(s)
- ineligibility (either arising during the study or retrospectively having been overlooked at screening)
- lost to follow-up

As data is collected on enrolled infants during their hospital stay, we anticipate minimal loss to follow- up. In the event of transfer of a patient to a regional centre following discharge from the tertiary unit, we will ensure 3 documented attempts are made to contact any subject lost to follow-up. Parents have the right to withdraw consent for participation in the study at any stage and this will not affect their infant's access to the best available treatment and care at the birth hospital or other hospital to which they might have been referred.

All subjects who discontinue should comply with protocol specified follow-up procedures. The only exception to this requirement is when a subject withdraws consent for all study procedures.
If a subject is withdrawn before completing the study, the reason for withdrawal must be entered on the appropriate case report form (CRF) page.
If a subject is withdrawn due to an adverse event, the investigator will arrange for follow-up visits until the adverse event has resolved or stabilised.

12 TREATMENT OF TRIAL SUBJECTS

12.1 Description of study treatment(s)

Surfactant (Curosurf, Chiesi Farmeceutici, Parma, Italy) is a white suspension. Each mL of suspension contains 80mg poractant alfa (surfactant extract) that includes 76mg of phospholipids and 1mg of protein of which 0.45mg is SP-B and 0.59mg is SP-C.
There are two vials of Curosurf: 1.5ml vial (contains 120mg poracant alfa) or 3ml vial (240mg poractant alfa).
The 120mg vial will be given to infants <26weeks gestation and a full 240mg vial to infants 26-28 weeks gestation.
The surfactant will be warmed by the clinicians prior to being drawn up in a sterile syringe as per manufacturer's recommendation. This will be done by opening the mouth gently and injecting the surfactant as a single bolus into the oropharynx using a syringe without a needle attached. This will be done as soon as possible after delivery, ideally before the umbilical cord has been clamped.

The dosing recommendations for initial treatment with Curosurf when given by endotracheal tube are:

- 200mg/kg for estab shed RDS
- 100 – 200mg/kg for prophylaxis

One or two further doses of 100mg/kg Curosurf may be given to infants who have persistent respiratory distress despite treatment with surfactant (maximum recommended dose 400mg/kg).

Infants in our study will not be weighed prior to enrolment. The 50th centile for birth weight (BW) for boys and girls according to gestational age (GA) is shown below

GA (weeks)	G r s BW (kg)	Boys BW (kg)
23	0.550	0.600
24	0.650	0.700
25	0.775	0.800
26	0.850	0.900
27	0.975	1.050

28	1.100	1.150

In our study, infants < 26 weeks will receive a full 120mg vial of Curosurf. We estimate that this will provide dosing in the therapeutic range as indicated below:

GA (weeks)	G r s BW (kg)	Dose (mg/kg)	Boys BW (kg)	Dose (mg/kg)
23	0.550	218	0.600	200
24	0.650	185	0.700	171
25	0.775	155	0.800	150

In our study, infants 26–28 weeks will receive a full 240mg vial of Curosurf. We estimate that this will provide dosing in the therapeutic range as indicated below:

GA (weeks)	G r s BW (kg)	Dose (mg/kg)	Boys BW (kg)	Dose (mg/kg)
26	0.850	282	0.900	267
27	0.975	246	1.050	229
28	1.100	218	1.150	209

12.2 Formulation, packaging and handling

CUROSURF (poractant a fa), manufactured by and censed from Ch es Farmaceut c , S.p.A., Parma, Ita y, 43100.
The suspension is available in sterile, rubber-stoppered clear glass vials containing:
1.5ml (120mg poractant alfa (surfactant extract)) of suspension: NDC Number 10122-510-01
3ml (240mg poractant alfa (surfactant extract)) of suspension: NDC Number 10122-510-03

The CUROSURF used for the trial (for all sites except Swedish sites) will be contained in individual ziplock bags and will have a sticker label on it containing the required clinical trial label. CUROSURF will be labelled in line with the recommendations in Annex 13 of the European Directive on Good Manufacturing Practice in clinical trials conducted with marketed investigational products.

Specifically, for sites conducting the trial in Sweden only, the Curosurf used in the trial will be the same as used routinely in the NICU. The Curosurf will be prescribed as per clinical routine and is only administered by appropriate health personnel. Due to the nature of the IMP handling, the Curosurf used in the study will have the commercial label but no study specific label, in line with LVFS 2011:19.

12.3 Storage and disposition of study treatment(s)

The suspension is stored in a refrigerator at +2 to +8 degrees Celsius, protected from light. Vials are for single use only. After opening the vial the unused portion is discarded.
The refrigerator temperature must be recorded on a temperature log on a daily basis to record proper function.
Curosurf is used routinely in the NICU via endotracheal administration. The suspension of Curosurf for use in this study is no different than that used routinely. However rather than administrating the medication endotracheally, it will be given via the oropharynx. Due to the emergent nature of some of these deliveries, the Curosurf used will be stored in the refrigerator in the NICU as per routine practice however will be separate for specific use for the POPART study (labelling on the IMP for all sites except Swedish sites). The study treatment will be stored and locked in a secure place until they are dispensed for use and the supply used for the trial will only be used within the context of this study.

12.4 Accountability of the study treatment

The study medication will be supplied by Chiesi Farmeceutici. The investigator is responsible for the control of the treatment(s) under investigation. Adequate records for the receipt and disposition of the IMP must be maintained.
The investigator will use a standard prescription form and the investigator/research nurse will collect the medication from the pharmacy as per routine practice in the country.

In our study, the ntervent on of OROPHARNYGEAL SURFACTANT s g ven as a once-off dose at b rth. Accountability and compliance with study treatments will be assessed by maintaining dispensing and return records.

12.5 Assessment of compliance

Compliance with study medications will be assessed through documentation at delivery in clinical notes and the study medications packaging will be stored post administration.
The investigator is responsible for ensuring that the study treatment is administered in compliance with the protocol. The intervention of 'OROPHARNYGEAL SURFACTANT' is given as a once-off dose at birth, thus compliance at later dates is not an issue.

12.6 Overdose of study treatment

The overdose of a study treatment will be handled in the same way as would any overdose of a medication. There have been no reports of over-dosage following the administration of Curosurf.

12.7 Prior and concomitant therapy

Any medication, other than the study medication taken during the study will be recorded in the CRF.

All participants will be newly-born infants, and so will not have any prior therapies.
We will record medication other than the study medication taken during the study in the CRF as follow:

- Postnatal corticosteroids for ventilator dependence
- Medical treatment for a patent ductus arteriosus
- Medical management of Necrotising enterocilitis (≥ Bell's stage 2)
- Intravitreal injection for retinopathy of prematurity

12.7.1 Permitted medications/non- investigational medicinal products

The only medications which will be used during the study are those medications that are routinely used on preterm babies in NICU.

12.7.2 Prohibited medications

There are no contraindicated medications for use in this study.

13 SAFETY REPORTING

Adverse events (AEs) will be recorded throughout the study and will be evaluated and classed according to the definitions below (European Directive 2001/20/EC). All adverse events will be collected and recorded in accordance with the detailed guidance on the collection, verification and presentation of adverse event/reaction reports arising from clinical trials on medicinal products for human use ('CT-3') while referencing the protocol specific guidance included in Section 13.3.

13.1 Definitions

13.1.1 Adverse event (AE)

Any untoward medical occurrence in a patient or clinical trial subject administered a medicinal product and which does not necessarily have a causal relationship with this treatment.
An adverse event can therefore be any unfavourable and unintended sign (including an abnormal laboratory finding, for example), symptom or disease temporally associated with the use of a medicinal product, whether or not considered related to the medicinal product

13.1.2 Adverse react on (AR)

All untoward and unintended responses to a medicinal product related to any dose.
The phrase 'responses to a medicinal product' means that a causal relationship between a study medication and an AE is at least a reasonable possibility, i.e., the relationship cannot be ruled out.
All cases judged by either the reporting medically qualified professional or the sponsor as having a reasonable suspected causal relationship to the study medication qualify as adverse reactions.

13.1.3 Ser ous adverse event

Any untoward medical occurrence or affect that at any dose:

- results in death,
- is life-threatening*,
- requires hospitalisation or prolongation of existing hospitalisation,
- results in persistent or significant disability or incapacity,
- is a congenital anomaly or birth defect
- important medical events**

*Regarding a life-threatening event, this refers to an event in which the subject was at risk of death at the time of the event; it does not refer to an event which hypothetically might have caused death if it were more severe.

**Some medical events may jeopardise the subject or may require an intervention to prevent one of the above characteristics/consequences. Such events (hereinafter referred to as 'important medical events') should also be considered as 'serious' in accordance with the definition

13.1.4 Severe adverse events

The term 'severity' is used here to describe the intensity of a specific event. This has to be distinguished from the term 'serious'.

13.1.5 Suspected unexpected ser ous adverse react ons

An adverse reaction, the nature or severity of which is not consistent with the applicable product information (e.g. investigator's brochure for an unauthorised investigational medicinal product or summary of product characteristics for an authorised medicinal product.

13.2 Evaluation of AEs and SAEs

Ser ousness, causa ty, sever ty and expectedness shou d be eva uated.

13.2.1 Assessment of ser ousness

The investigator should make an assessment of seriousness as defined in section 13.1.

13.2.2 Assessment of casua ty

All adverse events judged by either the investigator or the sponsor as having a reasonable suspected causal relationship to an investigational medicinal product qualify as adverse reactions.
The causality assessment given by the investigator should not be downgraded by the sponsor.
The investigator/sponsor must make an assessment of whether the AE/SAE is like y to be re ated to treatment according to the following definitions:

<u>Unrelated</u>
Where an event is not considered to be related to the study medication.

<u>Possibly</u>
Although a relationship to the study medication cannot be completely ruled out, the nature of the event, the underlying disease, concomitant medication or temporal relationship make other exp anat ons poss b e.

<u>Probably</u>
The temporal relationship and absence of a more likely explanation suggest the event could be related to the study medication.

A l AEs/SAEs judged as having a reasonable suspected causal relationship (e.g. possibly, probably) to the study med cat on w be cons dered as ARs/SARs.

A l AEs/SAEs judged as be ng re ated (e.g. possibly, probably) to an interaction between the study medication and another medication will also be considered to be ARs/SAR.

Alternative causes such as natural history of the underlying disease, concomitant therapy, other risk factors and the temporal relationship of the event to the treatment should be considered.

13.2.3 Assessment of sever ty

The investigator will make an assessment of severity for each AE/SAE and record this on the CRF according to one of the following categories:

<u>Mild</u>
An event that is easily tolerated by the subject, causing minimal discomfort and not interfering with every day activities.

<u>Moderate</u>
An event that is sufficiently discomforting to interfere with normal everyday activities.

<u>Severe</u>
An event that prevents normal everyday activities.

Note: the term ‘severe’, should not be confused with ‘serious’ which is a regulatory definition based on subject/event outcome or action criteria

13.2.4 Assessment of expectedness

The expectedness of an adverse reaction will be determined by the sponsor according to the reference document e.g. the investigator's brochure for a non-authorised investigational medicinal product, or the summary of product characteristics for an authorised medicinal product which is used according to the terms and conditions of the marketing authorisation. For CUROSURF the summary of product characteristics will be used as the reference safety information for assessment of expectedness of an adverse react on.

13.3 Reporting procedures for all adverse events

The investigational medicinal product (IMP), Curosurf, is licensed for use for prevention and treatment of respiratory distress syndrome (RDS) in premature infants in Europe for more than 25 years. It is given as a single dose by the endotracheal route and may on occasion be repeated. It has an excellent safety profile.

Many of the adverse events which are listed in the Summary of Product Characteristics for Curosurf (portactant alfa) below occur commonly in preterm infants who are admitted to the Neonatal Intensive Care Unit (NICU), whether or not they have received surfactant. Others are seen infrequently in this population, whether or not they have received surfactant.

System organ Class	Adverse Reaction	Frequency
Infections and infestations	Sepsis	Uncommon
Nervous system disorders	Haemorrhage intracranial	Uncommon
Cardiac disorders	Bradycardia	Rare
Vascular disorders	Hypotension	Rare
Respiratory, thoracic and mediastinal disorders	Bronchopulmonary dysplasia	Rare
	Pneumothorax	Uncommon
	Pulmonary haemorrhage	Rare
	Hyperoxia	Not known
	Cyanosis neonatal	Not known
	Apnoea	Not known
Investigations	Oxygen saturation decreased	Rare
	Electroencephalogram abnormal	Not known
Injury, poisoning and procedural complications	Endotracheal intubation complication	Not known

AEs that are directly related to the underlying condition of extreme prematurity do not need to be reported as an AE on this study, for example:

1. Bradycard a, hypotens on, hyperox a, cyanos s, apnoea, oxygen desaturat ons
   These events frequently occur several times per day in this population, whether or not they have received surfactant. Most often these events are short in duration and not of clinical significance. Regular reporting of these adverse events in the CRF is not required.

2. Intracranial haemorrhage
   Some degree of intracranial haemorrhage is detected in ≥30% of infants in this population, whether or not they have received surfactant. Regular reporting of these adverse events in the CRF is not required. All enrolled infants are being screened for intracranial haemorrhage; it is being recorded in the CRF and measured as a secondary outcome.

3. Bronchopu monary dysp as a
   Bronchopulmonary dysplasia occurs in up to ≥50% in this population of infants, whether or not they have received surfactant. Regular reporting of these adverse events in the CRF is not required. All enrolled infants are being screened for bronchopulmonary dysplasia; it is being recorded in the CRF and measured as a secondary outcome.

4. Pneumothorax, pu monary haemorrhage
   These are rare events in this population of infants, whether or not they have received surfactant. Regular reporting of these adverse events in the CRF is not required. All enrolled infants are being screened for pneumothorax and pulmonary haemorrhage; it is being recorded in the CRF and measured as a secondary outcome.

5. Endotracheal intubation complication
   Endotracheal intubation complication is not relevant as we are not giving surfactant via this route of administration as the intervention arm for this study. Regular reporting of these adverse events in the CRF is not required.

Only AEs which are not directly associated to the underlying condition of extreme prematurity should be reported. The following information will be recorded: description, date of onset and end date, severity, assessment of relatedness to the study medication, other suspect medication or device and action taken. Follow-up information should be provided as necessary.

AEs considered related to the study medication as judged by an investigator or the sponsor will be followed until resolution or until the event is considered stable. All related AEs that result in a subject's withdrawal from the study or are present at the end of the study, should be followed up until a satisfactory resolution occurs.

It will be left to the investigator's clinical judgment whether or not an AE is of sufficient severity to require the subject's removal from the study. If this occurs, the subject must undergo an end-of-study assessment and be given appropriate care under medical supervision until symptoms cease or the condition becomes stable.

The severity of events will be assessed on the following scale: mild, moderate, severe.

The relationship of AEs to the study medication will be assessed by the investigator.

13.4 Reporting procedures for serious adverse events

As the infants involved in this study are premature, their underlying condition will lead to many adverse events which could be deemed 'Serious' due to meeting the criteria of:

- is life-threatening*,

- requires hospitalisation or prolongation of existing hospitalisation,
- results in persistent or significant disability or incapacity,
- is a congenital anomaly or birth defect

For the purposes of this study, only events which meet the criteria below should be reported as Serious Adverse Events:
- results in death, or
- important medical events**

*Regarding a life-threatening event, this refers to an event in which the subject was at risk of death at the time of the event; it does not refer to an event which hypothetically might have caused death if it were more severe.

**Some medical events may jeopardise the subject or may require an intervention to prevent one of the above characteristics/consequences. Such events (hereinafter referred to as 'important medical events') should also be considered as 'serious' in accordance with the definition.

The investigator will report all serious adverse events immediately to the sponsor except for those that the protocol or investigator's brochure identifies as not requiring immediate reporting. The immediate report will be followed by detailed, written reports. The immediate and follow-up reports will identify subjects by unique code numbers assigned to the latter.

The immediate report will be made by the investigator within a very short period of time and under no circumstances should this exceed **24 hours** following knowledge of the serious adverse event.

All SAE information must be recorded on an SAE forms and sent expeditiously to the sponsor to the specific pharmacovigilance team at the SAE reporting email address: sae.reporting@ucd.ie where they will be received and processed. Additional information received for a case (follow-up or corrections to the original case) need to be detailed on a new SAE form and sent expeditiously to the sponsor.

The sponsor will keep detailed records of all adverse events which are reported to him by the investigator or investigators.

In cases where reporting is not required immediately the investigator will report within the appropriate time frame, taking account of the specificities of the trial and of the serious adverse event, as well as possible guidance in the protocol or the Summary of product characteristics.

The sponsor will report all SUSARs to the competent authorities and the ethics committees concerned. Fatal or life-threatening SUSARs must be reported within **7 days**. SUSARs which are not fatal and not life-threatening are to be reported within **15 days**. The sponsor will also inform all investigators concerned of relevant information about SUSARs that could adversely affect the safety of subjects.
If the initial report is incomplete, e.g. if the sponsor has not provided all the information/assessment within seven days, the sponsor will submit a completed report based on the initial information within an additional eight days.

If significant new information on an already reported case is received by the sponsor, the clock starts again at day zero, i.e. the date of receipt of new information. This information will be reported as a follow-up report within **15 days.**

In addition to the expedited reporting above, the sponsor shall submit once a year throughout the clinical trial or on request, a safety report to the competent authority and ethics committees. The annual safety report will be presented in the DSUR format as per ICH guideline E2F - Note for guidance on development safety update reports. This is a legal requirement.

13.5 Data Safety Monitoring Board (DSMB)

A DSMB will be established to perform ongoing safety surveillance and to perform interim analyses on the study data. The DSMB will be an independent committee, composed of a minimum of three members; at least two will be clinicians not involved in the trial but with experience and expertise in clinical trials; at least one member will be a clinician with expertise in neonatology. Each member will state that they have no conflict of interest with the sponsor or company involved in the study or any other conflicting interest to declare.

The DSMB will meet on a 6-monthly basis after start of the trial and will review the frequency and severity of adverse events in both treatment groups. If they observe any significant excess of serious adverse events in the intervention group associated with the intervention, they may recommend premature termination of the trial on the basis of serious safety concerns.

The DSMB will also conduct interim analysis to determine whether the data provide overwhelming evidence of efficacy or futility, defined as a highly statistically significant difference (i.e. $P < 0.001$) in the primary outcome or a highly statistically significant difference in the important secondary outcome of death before hospital discharge. Interim analysis for efficacy or futility will be carried out after approximately 50% of participants (n=126) have completed the study. The DSMB may recommend early termination of the trial due to efficacy or futility.

The advice(s) of the DSMB will be notified upon receipt by the sponsor to the REC and CA that approved the protocol. With this notification a statement will be included indicating whether the advice will be followed.

14 STATISTICS

14.1 Description of statistical methods

A Statistical Analysis Plan (SAP) will be written by the trial statistician. The SAP will detail the implementation of all of the planned statistical analysis in accordance with the main features stated in this section of the protocol and amendments if applicable. The SAP must be finalized before the database is locked, which is required to commence the study analysis.

14.2 Determination of sample size subjects

In randomised studies, the rate of mechanical ventilation in the days after birth among preterm infants treated with CPAP alone has varied from 46– 60%.(2, 11, 19-21) Minimally invasive surfactant techniques were shown to reduce the rate of mechanical ventilation to from 46% to 28% in a cohort of infants with a gestational age between 26 and 28 weeks. (21)

Sample size for this study was calculated in G*power, using a two-sided, two-proportion Z test. To detect a difference in the rate of endotracheal intubation for respiratory failure of 18% between treatment arms (assuming a rate of 46% in the control group and 28% in the intervention arm) with 80% power and a 5% significance level, a sample size of 224 infants would be required. To compensate for a death rate of 10% (estimated from local data, The National Maternity Hospital, Neonatal Clinical Report for 2015) before the primary outcome can be determined (i.e. at 120 hours), we will need to recruit a total of **250** infants to this study.

The National Maternity Hospital is a stand-alone university maternity hospital with a tertiary NICU to which > 150 infants < 1500g are admitted annually. We have a track record of initiating, conducting, completing, presenting and publishing investigator-led randomised controlled trials, including trials that compared delivery room interventions,(12-14) trials that had respiratory failure in the NICU as the primary outcome(15) and trials that compared investigational medicinal products(16) in preterm infants. Though the enrolment rates to our studies amongst eligible infants are consistently excellent (>80%), we believe it will be necessary to enrol infants at multiple sites in order to enrol our planned target sample of 250 infants in a timely fashion. We have a track record enlisting the help of collaborators

nationally(16) and internationally(14, 17) to perform our studies. We believe that with their help, we can enrol these infants in 2–3 years.

14.3 Analysis sets

This table defines the sets of subjects whose data are to be included in the statistical analyses.

Full Analysis Set (Intention-To-Treat principle)	All randomized patients
Per Protocol Set	Patients in the Full Analysis Set, excluding those with relevant protocol deviations which could affect the evaluation of primary endpoint. The Per Protocol set will exclude:<br>• Infants in the intervention arm who did not receive study treatment<br>• Infants with incomplete data on the primary endpoint
Safety Set	Patients in the intervention arm who received prophylactic surfactant and all patients in the control arm (for comparison)

Efficacy analysis will be carried out following the Intention-To-Treat (ITT) principle. Per protocol analysis of the primary endpoint will also be carried out for sensitivity analysis. Safety data will be analysed using the safetyset.

14.4 Demographic and baseline disease characteristics

Demographic and baseline data will be summarized for each treatment group. The date of birth, gender and race of the infant will be recorded. Gestation, multiplicity, birth weight and resuscitation at delivery, including Apgar scores, will be documented. Maternal age will be recorded. We will also record administration of antenatal steroids, preterm premature rupture of membranes (evidence of and duration of), intra-partum antibiotic use, the mode of delivery and indication for mode of delivery.

Descriptive analysis

The following summary statistics will be presented to summarize demographic and baseline disease characteristics by treatment arm:

- quantitative variables will be summarized by: number of observed and missing values, mean, median, standard deviation, IQR, minimum and maximum;
- categorical variables will be summarized by: number of observed and missing values, frequencies per category, percentages per category

14.5 Efficacy analysis

Analysis of efficacy endpoints will be carried-out following the Intention-To-Treat (ITT) principle. A Per Protocol analysis will also be carried out on the primary endpoint for sensitivity analysis. For all statistical analysis, a p-value < 0.05 will be considered statistically significant.

Interim analysis of the primary endpoint and the important secondary endpoint, death before hospital discharge, will be performed by an external data safety monitoring board. This will be performed once half the planned sample (126 participants) have completed the trial.

14.5.1 Primary efficacy endpoint

For the primary endpoint, the incidence of endotracheal intubation for respiratory failure within 120 hours of birth, the following analysis will be carried out.

- Descriptive statistics will be calculated to compare the rate of endotracheal intubation across treatment arms. Ratios of relative risk will be presented with 95% confidence intervals.
- A superiority hypothesis test (two-sided, two-proportion Z test) will be carried out to investigate whether the rate of endotracheal intubation differs between intervention and standard-of-care. In this analysis, infants intubated for signs of respiratory failure (whether or not they strictly met the pre-defined criteria in the protocol) will be considered to have met the primary endpoint.
- A competing risks model will be applied to investigate the effect of the intervention on the primary endpoint, adjusting for other outcomes that may impact on observation of the primary endpoint (e.g. death occurring before 120 hours of birth or intubation prior to 120 hours without meeting the criteria for respiratory failure defined in the protocol).
- Regression modelling will assess the effect of the intervention on the occurrence of endotracheal intubation within 120 hours of birth, adjusting for measured covariates of interest, including centre, gestational age, birth weight, gender, mode of delivery (vaginal versus caesarean birth) and antenatal corticosteroid treatment, to determine whether the estimated intervention effect is sensitive to these covar ates.

14.5.2 Secondary eff cacy endpo nts

Each categorical secondary endpoint will be analysed with the following approach.

- Descriptive statistics will be calculated to compare the rate of occurrence of the endpoint across treatment groups (frequency, percentage). Ratios of relative risk will be presented with 95% conf dence nterva s.
- A superiority hypothesis test (two-sided, two-proportion Z test) will be carried out to investigate whether the rate of occurrence of the endpoint differs between intervention and standard-of-care.

For the important secondary endpoint of death before hospital discharge, regression analysis will be employed to determine sensitivity of the estimated intervention effect to potentially relevant covariates; including centre, gestational age, birth weight, gender, mode of delivery (vaginal versus caesarean birth) and antenatal corticosteroid treatment.

Each quantitative secondary endpoint (e.g. duration of mechanical ventilation, duration of hospitalisation), will be analysed using the following approach.

- Descriptive statistics will be calculated to compare the endpoint across treatment groups (mean, median, standard deviation, IQR, min, max). The difference in means (or medians where relevant) between treatment groups will be presented with a 95% confidence interval.
- A superiority hypothesis test (two-sided independent sample t-test) will be carried out to test for a difference in means between control and intervention. Alternatively, if t-test assumptions are not valid, a Mann-Whitney U test will be carried out to test for a difference in the the endpoint between control and intervention.

14.5.3 Subgroup ana ys s

Subgroups of interest include infants of different gestational age strata (e.g. less than 26 weeks, and 26-28 weeks gestation at birth), and infants from different participating centres. Subgroup analysis of the primary outcome and the important secondary outcome of death before hospital discharge will be carried out for these subgroups by:

- Summarizing the frequency and rate of the outcome by treatment arm, for each subgroup seperately
- Regression modelling of the outcome evaluating interaction effects for treatment type and centre, and for treatment type and gestational age

14.6 Safety analysis

Adverse events following administration of oropharyngeal surfactant will be documented e.g. bradycardia, desaturation. Should an infant require endotracheal intubation for respiratory failure, the intubation difficulty and attempts after oropharyngeal administration of surfactant will also be recorded.

Safety analyses will be carried-out on patients in the Safety Set, the definition of which will be finalized n the Stat st ca Ana ys s P an (SAP).

Adverse events

The number of adverse events (i.e. all events occurring, worsening or becoming serious after the randomisation), and the number and percentage of infants reported as having at least one emergent adverse event, will be reported by system organ class and preferred term, for each treatment group.

The same description will be performed for serious adverse events (SAE), severe AE, AE treatment related and AE leading to IMP withdrawal.

14.7 The level of statistical significance

The type I error rate for interim analysis will be set to 0.001 in accordance with the Haybittle-Peto stopping boundary. For final analysis, the type I error rate will be set at 0.05. Confidence intervals will be reported at a 95% confidence level.

14.8 Criteria for the termination of the trial

Interim analysis of the primary outcome and the important secondary outcome of death before hospital discharge will be performed on half of the planned sample (126) by a DSMB. The Haybittle-Peto boundary will be used as a stopping rule. Therefore, the DSMB may recommend terminating the study early for large and statistically significant differences ($p<0.001$) between groups in the primary outcome or in the important secondary outcome of death before hospital discharge; or for unanticipated concerns regarding the safety of enrolled infants.

14.9 Procedure for accounting for missing, unused and spurious data

All data will be checked for missing values and followed-up to obtain data where possible. Missing data will be summarized by variable and by treatment group.

Missing values will be checked for processes possibly affecting missing data i.e. non-MCAR (Missing Completely at Random) processes correlated with outcomes. The method for handling missing data will then be specified accordingly in the Statistical Analysis Plan (SAP) before the database is locked.

Spurious or anomalous data will also be queried and corrected if found to be incorrect. No data will be excluded as outliers. However, statistical methods (rank-based methods) may be used to describe the data if outliers exist and adversely affect statistical analysis.

In compliance with the ethical conduct of RCTs, no data collected will be unused: all data will be described.

14.10 Procedure for reporting any deviation(s) from the original statistical plan

Any deviation(s) from the original Statistical Analysis Plan should be described and justified in the final report.

15 DIRECT ACCESS TO SOURCE DATA/DOCUMENTS

Direct access will be granted to authorised representatives from the sponsor, host institution and the regulatory authorities to permit trial-related monitoring, audits and inspections.

16 DATA HANDLING AND RECORD KEEPING

A data capture system is going to be used for this study. A case report form (CRF) is designed to record the data required by the protocol and collected by the investigator in the patient's clinical source

documents. Source documents for this study will include hospital records and procedure reports and data collection forms.
Data will be coded. It will be stored in a locked filing cabinet then uploaded onto a password-protected computer in a locked office.

16.1 Data collection, source documents and case report forms (CRF)

Source documents for this study will include hospital records and procedure reports and data collection forms. These documents will be used to enter data on the CRFs.. Participants who are transferred to another hospital before discharge home may have the following data points outstanding at the time of their transfer:

- o Death before hospital discharge
- o Survival without BPD at hospital discharge
- o Survival without CLD at hospital discharge
- o Duration of hospitalisation
- o Use of home oxygen therapy

The PI at the study site, or their delegate(s), will collect outstanding data in consultation with colleagues at the destination hospital and enter it in the participant's record at the study site. This data will be used to populate the CRF.
All data entered on CRFs must be entered legibly. If an error is made, the error will be crossed through with a single line in such a way that the original entry can still be read. The correct entry will then be clearly inserted, and the alterations will be initialled and dated by the investigator. Data reported on the CRF that are derived from source documents must be consistent with the source documents or the d screpanc es must be exp a ned.
All documents will be stored safely in confidential conditions. On all study-specific documents other than the signed consent, the subject will be referred to by the study subject indenisation number/code.

16.2 Data reporting

Data are collected via paper CRF initially and then transferred to a computer to be stored in a secure database. Subjects will be identified by a code in the database. The name and any other identifying detail will not be included in any study data electronic file.
For data collected in the CRF, the sponsor is responsible for data processing including data validation and coding performed according to a specification manual that describes the checks to be carried out. As a result of data validation, data may require some changes. An electronic data clarification form is sent to the investigator who is required to respond to the query and make any necessary changes to the data.
When data validation is achieved, a blind review of the data is performed according to the sponsor standard operating procedure. When the database has been declared to be complete and accurate, it will be locked and made available for data analysis.

17 RETENTION OF ESSENTIAL DOCUMENTS

Essential documents will be retained until at least 15 years after the publication of the clinical study report. These documents should be retained for a longer period however if required by the applicable regulatory requirements or by an agreement with the sponsor.
The investigator/institution should agree to retain the trial-related essential documents as required by the applicable regulatory requirements and until the sponsor informs the investigator/institution these documents are no longer necessary.

18 QUALITY CONTROL AND QUALITY ASSURANCE PROCEDURES

This study will examine the off-label use of a licensed product. Curosurf is licensed for the treatment of infants born at 24 – 31 weeks gestation who are at risk of RDS; however, it is not licensed for oropharyngeal administration nor for infants born at < 24 weeks gestation that are offered intensive care who may be enrolled to this study. This study will thus be classed as a trial of an Investigational Medicinal Product (IMP) and will be subject to the relevant legislation and regulated by the competent authorities [e.g. Health Products Regulatory Authority (HPRA, https://www.hpra.ie/) in Ireland] in the

jurisdictions where the trial is performed. Standard procedures for reporting adverse event will be used n accordance w th Good C n ca Pract ce (GCP) gu de nes.

The investigator agrees, when signing the Study Protocol, to adhere to the instructions and procedures described in it and to the principles of GCP to which it conforms. The regulatory permission to perform the study will be obtained in accordance with applicable regulatory requirements. All ethical and regulatory approvals must be available before a patient is exposed to any study-related procedure, including screening tests to determine eligibility.
The investigator will allow the monitor to visit the site and facilities where the study will take place in order to ensure compliance with the protocol requirements and ICH GCP.
Training sessions may be organised for the investigators and/or instruction manuals may be given to the study team as required.

In addition, interim analysis of primary outcome and selected secondary outcome data will be performed on half the planned sample (126) by an external data safety monitoring board. They may recommend terminating the study early for large and statistically significant differences (e.g. $P < 0.01$) between the groups in the primary outcome that demonstrate efficacy; or for unanticipated concerns for the safety of enrolled infants.

19 AUDITS AND INSPECTIONS

This trial may be subject to internal or external auditing or inspections procedure to ensure adherence to GCP. Access to all trial-related documents will be given at that time.
A quality assurance audit may be conducted by the sponsor or its agent at any time during, or shortly after, the study. The investigator will permit an independent audit by an auditor mandated by Sponsor, after reasonable notice. The purpose of an audit is to confirm that the study is conducted as per protocol, GCP and applicable regulatory requirements, that the rights and well-being of the patients enrolled have been protected, and that the data relevant for the evaluation of the investigational medicinal product have been captured, processed and reported in compliance with the planned arrangements. The investigator will permit direct access to all study documents, drug accountability records, medical records and source data.
Regulatory authorities may perform an inspection of the study up to several years after its completion. If an inspection is announced the Sponsor will be informed immediately

20 ETHICS

Ethical approval will be sought by each participating centre. Approval will be obtained prior to commencement of the trial and in compliance with Ethics committee's requirements.

20.1 Declaration of Helsinki

The sponsor will ensure that this study is conducted in accordance with the ethical principles that have their origins in the Declaration of Helsinki.

20.2 Good Clinical Practice

This study will be conducted in accordance with Good Clinical Practice (GCP), as defined by the International Conference on Harmonisation (ICH) and in accordance with the ethical principles underlying European Union Directive 2001/20/EC and 2005/28/EC.

20.3 Approvals

Required documents including the protocol, informed consent form, subject information leaflet, investigational medicinal product dossier, investigators brochure and any other required documents will be submitted to a recognised research ethics committee and the competent authority for written approval.
The sponsor will submit and obtain approval from the above parties for substantial amendments to the original approved documents.

20.4 Informed consent

Prior to delivery a member of the research team or other senior doctor will approach parent(s)/guardian(s) of eligible infants to inform them about the study. The team member will explain the purpose and nature of the study and provide written information for the parent(s)/guardian(s) to keep. Parent(s)/guardian(s) will be offered the opportunity to have another family member or support person present while the study is explained. If English is not their first language, they will also be offered the opportunity to have an interpreter present while the study is explained.

Written consent for enrolment of the infant in the study will then be sought, where applicable, by both parents/guardians prior to any study-related activities, or as per local requirements and as approved by the ethics committee for the site. Parents will be informed that they may withdraw their child from the study at any time should they so wish; and that a decision not to consent to their infants participation in the study or to withdraw their infant from the study once enrolled will not affect their infants access to the best available treatment and care at the birth hospital or other hospital to which they might be referred. Consent forms will be kept securely and a copy will be provided to the parents for their records.

20.5 Benefits and risks assessment

We will perform this study to establish whether giving preterm infants surfactant into their oropharynx at birth reduces their need for subsequent intubation in the first 5 days of life.

Potential benefits of the intervention include:

- Easier method of surfactant administration
- Cheaper method of surfactant administration
- Less discomfort to infants during surfactant administration
- May reduce costs associated with ventilation
- May reduce adverse effects of ventilation

All babies in both groups will be closely watched to see if they need extra treatment for their RDS at any stage, including surfactant given endotracheally. The babies will be treated equally and given whatever treatment is necessary, whether or not they have already been given surfactant into the oropharynx.

Preterm babies who are intubated and receive mechanical ventilation are at risk of chronic lung disease of prematurity. Avoidance of mechanical ventilation and administration of surfactant through less invasive techniques has been an important area of research over the last number of years.

There is evidence from animal (22, 23) and human studies (4, 5) that suggest that pharyngeal administration of surfactant to newborn infants at risk of RDS is safe and may be effective. A recent Cochrane Review (24) highlighted the need for a well-designed randomised control trial to address this question.

An independent DSMB will be established to perform ongoing safety surveillance and to perform interim analyses on the study data. The DSMB will meet on a regular basis and will review the frequency and severity of adverse events in both treatment groups. If they observe any significant excess of serious adverse events in the intervention group associated with the intervention, they may recommend to prematurely terminate the trial on the basis of serious safety concerns. Interim analysis of the primary endpoint and the secondary endpoint death before hospital discharge will be performed by the DSMB. This will be performed once half the planned sample (126 participants) have completed the trial.

20.6 Subject confidentiality

The trial staff will ensure that the subjects' anonymity is maintained. The subjects will be identified only by initials and a subject's identification number on the CRF and any database. All documents will be stored securely. The study will comply with the Data Protection Act.

21 FINANCING AND INSURANCE/INDEMNITY

The UNIVERSITY COLLEGE OF DUBLIN s the SPONSOR and t w ensure that every nvest gator s covered by a Public Liability (‘negligent harm’) insurance that applies for the clinical trial. All investigators are qualified and practicing physicians and are thus insured by the clinical indemnity scheme (CIS).

The study will be funded by Chiesi Farmeceutici (Parma, Italy).

22 CLINICAL STUDY REPORT AND PUBLICATION POLICY

The clinical study report will be drafted in compliance with the regulatory requirements and Sponsor’s standard operating procedure.
The sponsor’s representative and the Chief Investigator must mutually agree on the final version. One copy of the final report will be dated and signed by the Chief Investigator and the Legal Representative of the Sponsor.

The responsibility for publication of the data obtained from this study lies with the study Chief Investigator. Neither the sponsor, nor any of the study funders, will have any involvement in data collection, data review, data analysis or preparation of the publication manuscript or in the decision to submit for publication.

23 REFERENCES

1. Rojas-Reyes MX, Morley CJ, Soll R. Prophylactic versus selective use of surfactant in preventing morbidity and mortality in preterm infants. The Cochrane database of systematic reviews. 2012(3):Cd000510.
2. Dargaville PA, Gerber A, Johansson S, De Paoli AG, Kamlin CO, Orsini F, et al. Incidence and Outcome of CPAP Failure in Preterm Infants. Pediatrics. 2016;138(1).
3. More K, Sakhuja P, Shah PS. Minimally invasive surfactant administration in preterm infants: a meta-narrat ve rev ew. JAMA Ped atr. 2014;168(10):901-8.
4. Ten centre trial of artificial surfactant (artificial lung expanding compound) in very premature bab es. Ten Centre Study Group. Br t sh med ca journa (C n ca research ed). 1987;294(6578):991-6.
5. Kattwinkel J, Robinson M, Bloom BT, Delmore P, Ferguson JE. Technique for intrapartum administration of surfactant without requirement for an endotracheal tube. J Perinatol. 2004;24(6):360-5.
6. Enhorning G, Shennan A, Possmayer F, Dunn M, Chen CP, Milligan J. Prevention of neonatal respiratory distress syndrome by tracheal instillation of surfactant: a randomized clinical trial. Pediatrics. 1985;76(2):145-53.
7. Merritt TA, Hallman M, Bloom BT, Berry C, Benirschke K, Sahn D, et al. Prophylactic treatment of very premature infants with human surfactant. N Engl J Med. 1986;315(13):785-90.
8. Hallman M, Merritt TA, Jarvenpaa AL, Boynton B, Mannino F, Gluck L, et al. Exogenous human surfactant for treatment of severe respiratory distress syndrome: a randomized prospective clinical trial. J Ped atr. 1985;106(6):963-9.
9. Lee K, Khoshnood B, Wall SN, Chang Y, Hsieh HL, Singh JK. Trend in mortality from respiratory distress syndrome in the United States, 1970-1995. J Pediatr. 1999;134(4):434-40.
10. Avery ME, Tooley WH, Keller JB, Hurd SS, Bryan MH, Cotton RB, et al. Is chronic lung disease in low birth weight infants preventable? A survey of eight centers. Pediatrics. 1987;79(1):26-30.
11. Finer NN, Carlo WA, Walsh MC, Rich W, Gantz MG, Laptook AR, et al. Early CPAP versus surfactant in extremely preterm infants. N Engl J Med. 2010;362(21):1970-9.
12. McCarthy LK, Molloy EJ, Twomey AR, Murphy JF, O'Donnell CP. A randomized trial of exothermic mattresses for preterm newborns in polyethylene bags. Pediatrics. 2013;132(1):e135-41.
13. McCarthy LK, Twomey AR, Molloy EJ, Murphy JF, O'Donnell CP. A randomized trial of nasal prong or face mask for respiratory support for preterm newborns. Pediatrics. 2013;132(2):e389-95.

14. http://www.isrctn.com/ISRCTN74486341?q=BREL&filters=&sort=&offset=1&tota Results=1&page=1&pageS ze=10&searchType=advanced-search [06.01.15].
15. Kieran EA, Twomey AR, Molloy EJ, Murphy JF, O'Donnell CP. Randomized trial of prongs or mask for nasal continuous positive airway pressure in preterm infants. Pediatrics. 2012;130(5):e1170-6.
16. https://www.clinicaltrialsregister.eu/ctr-search/search?query=2011-002962-19 [06.01.15].
17. http://www.isrctn.com/ISRCTN65161530?q=NORD&filters=&sort=&offset=4&tota Results=27&page=1&pageS ze=10&searchType=bas c-search [06.01.15].
18. http://www.chiesi.uk.com/system/file2s/20/original/Curosurf_CSP021-2.pdf?1286374254 [06.01.15].
19. Morley CJ, Davis PG, Doyle LW, Brion LP, Hascoet JM, Carlin JB. Nasal CPAP or intubation at birth for very preterm infants. N Engl J Med. 2008;358(7):700-8.
20. Dunn MS, Kaempf J, de Klerk A, de Klerk R, Reilly M, Howard D, et al. Randomized trial comparing 3 approaches to the initial respiratory management of preterm neonates. Pediatrics. 2011;128(5):e1069-76.
21. Gopel W, Kribs A, Ziegler A, Laux R, Hoehn T, Wieg C, et al. Avoidance of mechanical ventilation by surfactant treatment of spontaneously breathing preterm infants (AMV): an open-label, randomised, contro ed tr a . Lancet. 2011;378(9803):1627-34.
22. Enhorning G, Grossmann G, Robertson B. Pharyngeal deposition of surfactant in the premature rabbit fetus. Biol Neonate. 1973;22(1):126-32.
23. Enhorning G, Robertson B, Milne E, Wagner R. Radiologic evaluation of the premature newborn rabbit after pharyngeal deposition of surfactant. American journal of obstetrics and gynecology. 1975;121(4):475-80.
24. Abdel-Latif ME, Osborn DA. Pharyngeal instillation of surfactant before the first breath for prevention of morbidity and mortality in preterm infants at risk of respiratory distress syndrome. The Cochrane database of systemat c rev ews. 2011(3):Cd008311.

UCD School of Medicine
Scoil an Leighis UCD
ucd.ie/medicine

Statistical Analysis Plan (SAP)

PROPHYLACTIC OROPHARYNGEAL SURFACTANT FOR PRETERM INFANTS: A RANDOMISED TRIAL (THE POPART TRIAL)

Administrative Information

Trial registration number:	EudraCT number: 2016-004198-41
SAP version:	1.0
Protocol version:	Version 2, 19-Jul-2019
Chief Investigator:	Prof. Colm P.F. O'Donnell
Sponsor:	Prof. Peter Doran, UCD

Contributors

Name	Affiliation	Role in SAP writing (Author/Reviewer)
Marie Galligan	University College Dublin	Author
Colm O'Donnell	University College Dublin	Reviewer

Revision history

Revision	Justification	Timing
1.0	Initial Version	27/04/22

Approved by

Name	Affiliation	Study Role	Date and Signature
Marie Galligan	UCD	Trial Statistician/Author	
		Reviewer *(if applicable)*	
Colm O'Donnell	NMH	Chief Investigator	

Contents

1. Introduction 5

1.1 Background and rationale 5

1.2 Objectives 5

1.3 Endpoints 5

1.4 Estimands 7

1.4.1 Primary estimand 7

2. Study methods 8

2.1 Trial design 8

2.2 Framework for analysis 8

2.3 Statistical interim analyses and stopping guidance 9

2.4 Timing of final analysis 9

2.5 Timing of outcome assessments 9

3. Statistical principles 10

3.1 Confidence intervals and *P* values 10

3.2 Analysis populations 10

3.2.1 Full analysis set (FAS) 10

3.2.2 Per-protocol (PP) 10

3.2.3 Safety population 10

4. Trial Population 11

4.1 Screening data 11

4.2 Eligibility 11

4.3 Recruitment 11

4.4 Baseline patient characteristics 11

4.5 Adherence and protocol deviations 12

4.6 Withdrawal/follow-up 12

5. Analysis 13

5.1 Analysis methods 13

5.1.1 Primary analysis 13

5.1.2 Sensitivity analyses 14

5.1.3 Subgroup analyses 15

5.2 Interim analyses 15

5.2.1 Adjustment of Confidence intervals and p-values 15

5.3 Missing data 16

5.4 Safety evaluation 17

5.5 Statistical software 17

5.6 Quality control 17

6. Changes from the protocol 19

7. References 19

1. Introduction

1.1 Background and rationale

Many preterm infants develop respiratory distress syndrome (RDS), a condition characterised by a relative lack of surfactant. Endotracheal surfactant therapy revolutionised the care of preterm infants in the 1990s. However, supporting newborns with RDS with continuous positive airway pressure (CPAP) and reserving endotracheal surfactant for those who develop respiratory failure despite CPAP yield better results than intubating all infants for surfactant. Half of preterm infants born before 29 weeks gestation initially managed with CPAP are intubated for surfactant. Intubation is difficult to learn and associated with adverse effects. Surfactant administration into the oropharynx has been reported in preterm animals and humans and may be effective. We wished to determine whether giving oropharyngeal surfactant at birth reduces the rate of endotracheal intubation for respiratory failure in preterm infants within 120 hours of birth.

1.2 Objectives

The primary objective is to investigate the efficacy of prophylactic oropharyngeal surfactant for reducing the rate of endotracheal intubation, compared to no intervention in infants at risk of RDS.

The secondary objective is to investigate the efficacy of prophylactic oropharyngeal surfactant compared to no intervention for preventing some possible complications in premature infants at risk of RDS. We will also record a number of variables relating to prematurity, from enrolment into the trial until discharge home from hospital.

1.3 Endpoints

Primary endpoint

The primary endpoint is the incidence of endotracheal intubation for respiratory failure within 120 hours of birth.

Enrolled infants will be intubated for persistent apnoea and/or bradycardia in the DR, or for respiratory failure in the NICU defined as ≥ 2 of:

- Clinical signs – worsening tachypnoea; grunting; subcostal, intercostal and/or sternal recession
- Acidosis – pH < 7.2 on 2 blood gases (arterial or capillary) ≥ 30 minutes apart
- O_2–FiO_2 > 0.4 to keep SpO_2 ≥ 90% for > 30minutes
- PCO_2 > 9.0 kPa on 2 blood gases (arterial or capillary) ≥ 30 minutes apart
- Apnoea – recurrent apnoea treated with mask ventilation
  The frequency of blood gas monitoring is based on the clinical decision of the treating physician, as per routine practice.

Enrolled infants will only be intubated if they reach the pre-determined criteria for respiratory failure. Attending clinicians may give endotracheal surfactant via a thin catheter (LISA technique) or via an endotracheal tube (ETT). After giving endotracheal surfactant for the treatment of RDS, attending clinicians may attempt to extubate the babies immediately ("INSURE") or they may elect to ventilate the babies for a longer period. This is at their discretion.

Secondary endpoints:

- Intubation in the delivery room (dr_infant_intub.factor)
- Number of attempts taken to successfully intubate in the DR (dr_infant_intubation_attempts)
- Chest compressions in the DR (chest_compressions.factor)
- Adrenaline administration in the DR (adrenaline.factor)
- Rectal temperature on admission to the NICU (nicu_rectal_temp)
- NICU intubation – first intubation occurring in the NICU (first_intubation_nicu.factor)
- Surfactant use before death or hospital discharge
    - Number of doses, including total dose
    - Intra-tracheal surfactant received post-intervention (ett_postrandom.factor)
    - Doses of post-intervention surfactant (ett_postrandom_dose_no)
- Respiratory distress syndrome (resp_distress_syndrome.factor)
    - Clinical evidence of respiratory distress with radiological evidence (ground glass appearance on CXR)
- Incidence of pneumothorax (pneumothorax.factor)
    - Incidence of pneumothorax on CXR
- Incidence of pulmonary haemorrhage (pulm_haem.factor)
    - Clinical evidence of pulmonary haemorrhage
- Mechanical ventilation (mechanical_ventilation.factor)
- Days of mechanical ventilation (mech_vent_days)
- Use of postnatal corticosteroids for ventilator dependence (post_steroids.factor)
- Days of duration of respiratory support (endotracheal ventilation, high-frequency oscillatory ventilation, CPAP, heated humidified high-flow nasal cannula O_2,low flow nasal cannula O_2) (resp_supp_days)
- Bronchopulmonary dysplasia (BPD) –supplemental O_2 at 28 days of life (o2_d28.factor)

- Chronic lung disease of prematurity (CLD) – need for supplemental O_2 at 36 weeks corrected gestational age (CGA) determined by physiological oxygen reduction test (o2_36wks.factor)
- Medical treatment for a patent ductus arteriosus (pda_medtr.factor)
    - Administration of ibuprofen or paracetemol for PDA
- Surgical treatment for a patent ductus arteriosus (pda_surgery.factor)
- Proven necrotising enterocolitis (≥ Bell's stage 2) (proven_nec.factor)
- Incidence of Intraventricular haemorrhage (IVH) (any (ivh_any.factor) and severe (ivh_severe.factor): IVH grade ≥ 3)
    - Evidence on surveillance cranial ultrasounds performed regularly in NICU as standard of care

- Incidence of cystic periventricular leukomalacia (cystic_pvl.factor)
  - Evidence on surveillance cranial ultrasounds performed regularly in NICU as standard of care
- Retinopathy of prematurity treated with laser photocoagulation or intravitreal injections (retinopathy_tr_req.factor)
  - Evidence on surveillance ophthalmology review performed regularly in NICU as standard of care
- Death before hospital discharge (hospitalisation_death.factor)
- Survival without BPD at hospital discharge (without_bpd_at_discharge.factor)
- Survival without CLD at hospital discharge (without_cld_at_discharge.factor)
- Duration of hospitalisation (hospitalisation_duration)
- Use of home oxygen therapy (home_oxygen.factor)
  - Discharged home on oxygen therapy

These are clearly defined outcome measures and will be recorded during the infants hospital stay prior to discharge home from hospital. These outcome measures include complications of prematurity that can occur at any stage from birth until discharge home and thus specific timeframes for measurement is not applicable in all cases.

1.4 Estimands

1.4.1 Primary estimand

The primary estimand in this trial is the difference in the rates of endotracheal intubation for respiratory failure while alive within 120 hours of birth in future infants treated with prophylactic oropharyngeal surfactant compared to infants treated with the standard of care (no intervention). Two potential intercurrent events were identified that could impact on observation of the primary endpoint: (1) death prior to 120 hours without observation of the primary endpoint and (2) intubation for respiratory failure without meeting the pre-specified criteria defined in the protocol.

Intercurrent event	Strategy for handling intercurrent event
(1) Infants who died within 120 hours of birth without having met the primary endpoint	While alive strategy – data collected on the subjects while alive will be used to determine the primary endpoint, even if the observation period is shorter than 120 hours
(2) Infants intubated for respiratory failure within 120 hours of birth without	Treatment policy strategy – subjects will be considered to have met the primary endpoint if they are intubated for respiratory failure within 120 hours of life (regardless of whether or not the protocol definition was met.

meeting the criteria specified in the protocol	

2. Study methods

2.1 Trial design

This is an international, multicentre, randomised parallel group control trial. Infants will be randomized in a variable block randomization scheduled, stratified by centre and gestational age, to one of two arms: the investigational arm consisting of oropharyngeal surfactant and standard-of-care (CPAP) and the control arm consisting of standard-of-care (CPAP) only.

Infants born less than 29 weeks' gestational age will be included if the treating physician plans to offer intensive care. Infants will be excluded if infants have major congenital anomalies and if the treating physician does not plan to offer intensive care. Written informed consent will be obtained before delivery.

Figure 1: Study Schema

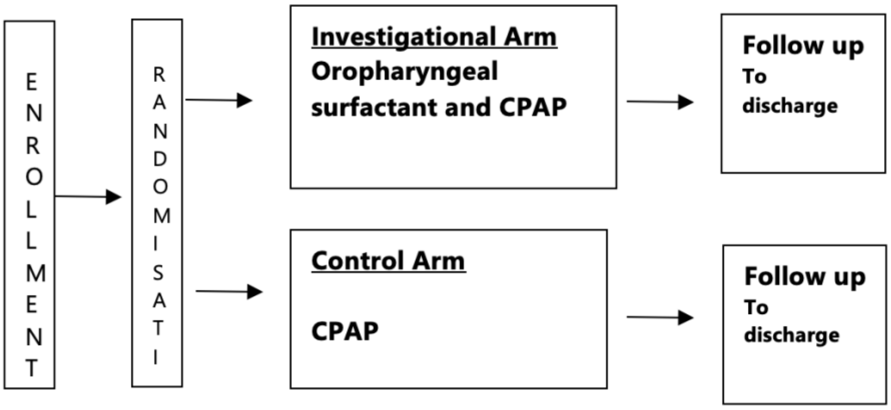

2.2 Framework for analysis

Analysis of efficacy endpoints will be carried-out following the Intention-To-Treat (ITT) principle. A Per Protocol analysis will also be carried out on the primary endpoint for sensitivity analysis. For all statistical analysis, a p-value < 0.05 will be considered statistically significant.

Interim analysis of the primary endpoint and the important secondary endpoint, death before hospital discharge, will be performed by an external data safety monitoring board. This will be performed once half the planned sample (126 participants) have completed the trial.

For the primary analysis of the primary endpoint, a superiority hypothesis test (two-sided, two-proportion Z test) will be carried out to investigate whether the rate of endotracheal intubation differs between intervention and standard-of-care. In this analysis, infants intubated for signs of respiratory failure will be considered to have met the primary endpoint.

2.3 Statistical interim analyses and stopping guidance

Efficacy interim analysis of the primary outcome and the important secondary outcome of death before hospital discharge will be performed on half of the planned sample (126 subjects) by a DSMB. The Haybittle-Peto boundary will be used as a stopping rule. Therefore, the DSMB may recommend terminating the study early for large and statistically significant differences (p<0.001) between groups in the primary outcome or in the important secondary outcome of death before hospital discharge; or for unanticipated concerns regarding the safety of enrolled infants.

The type I error rate for interim analysis will be set to 0.001 in accordance with the Haybittle-Peto stopping boundary. For final analysis, the type I error rate will be set at 0.05. Confidence intervals will be reported at a 95% confidence level.

2.4 Timing of final analysis

Final analysis will be carried out when all subjects reach the last study visit, data entry and validation is completed and the database is locked.

2.5 Timing of outcome assessments

Infants will be randomised at delivery. Infants randomised to oropharyngeal surfactant will receive a dose of surfactant. Infants randomised to the control group will not have anything injected into their oropharynx and will be stabilised on CPAP in the DR as per routine practice.

The primary endpoint is the incidence of endotracheal intubation for respiratory failure within 120 hours of birth. Other secondary outcomes have predefined outcome measures and will be recorded on the individual CRF during the course of hospitalisation.

3. Statistical principles

3.1 Confidence intervals and *P* values

The type I error rate for interim analysis will be set to 0.001 in accordance with the Haybittle-Peto stopping boundary. For final analysis, the type I error rate will be set at 0.05. Confidence intervals will be reported at a 95% confidence level.

3.2 Analysis populations

3.2.1 Full analysis set (FAS)

The Full Analysis Set (FAS) will include all trial subjects enrolled into the trial and randomised. The evaluation is carried out strictly in accordance with the allocation by randomization according to the intention-to-treat principle (ITT).

3.2.2 Per-protocol (PP)

The per-protocol (PP) population includes subjects in the Full Analysis Set, excluding those with relevant protocol deviations which could affect the evaluation of primary endpoint. The Per Protocol set will exclude:

- Infants in the intervention arm who did not receive study treatment
- Infants with incomplete data on the primary endpoint

3.2.3 Safety population

Patients in the intervention arm who received prophylactic surfactant and all patients in the control arm (for comparison)

4. Trial Population

4.1 Screening data

For each study centre, data will be collected on number of subjects screened, number eligible, number enrolled and reasons for screen failures. This data will be reported in the clinical study report (CSR) by study centre.

4.2 Eligibility

Inclusion and exclusion criteria are defined in the study protocol.

4.3 Recruitment

A CONSORT patient flow diagram will be constructed following the CONSORT 2010 standards (http://www.consort-statement.org/consort-2010) to illustrate number of subjects screened, enrolled and randomized.

4.4 Baseline patient characteristics

Evaluations of the baseline characteristics will be based on the FAS. They will be presented in a descriptive summary table by treatment group—continuous variables as mean and standard deviation or median and quartiles, and categorical variables as absolute and relative frequencies. No statistical comparisons of patient characteristics at baseline will be performed.

Maternal baseline characteristics to be summarized include

- Age
- Ethnicity
- In Utero antenatal transfer
- Administration of antenatal steroids (incidence and number of doses)
- Spontaneous labour
- Preterm premature rupture of membranes (evidence of and duration of),
- Intra-partum antibiotic use
- Mode of delivery and
- Indication for mode of delivery
- Administration of regional anaesthesia (e.g. spinal, epidural)
- Administration of general anaesthesia

Infant baseline characteristics to be summarized include

- Best estimate of gestational age (GA)
- Method for determining GA
- Gender
- Multiplicity
- Birth weight

- Apgar scores (1, 5, 10 min)
- resuscitation at delivery

4.5 Adherence and protocol deviations

Administration of the intervention at delivery will be documented, along with reason for non-administration for infants allocated to receive the intervention. The end of study form will document whether infants completed the study per protocol, with reasons for early discontinuation also recorded on the CRF. Protocol deviations will be summarized in the CSR.

4.6 Withdrawal/follow-up

All withdrawals and losses to follow-up will be listed with the time points and reasons (if available).

5. Analysis

5.1 Analysis methods

5.1.1 Primary analysis

The primary estimand in this trial is the difference in the rates of endotracheal intubation for respiratory failure while alive within 120 hours of birth in future infants treated with prophylactic oropharyngeal surfactant compared to infants treated with the standard of care (no intervention). The primary analysis will be done on the full analysis set according to the intention-to-treat principle. For infants who died within 120 hours of randomization, the primary endpoint will be documented for the time period in which they were alive. Infants who were intubated without (or prior to) meeting the definition of respiratory failure defined in the protocol will be included, and will considered to have met the primary endpoint in the main analysis.

Primary endpoint analysis

For the primary endpoint, the incidence of endotracheal intubation for respiratory failure within 120 hours of birth, the following analysis will be carried out.

- Descriptive statistics will be calculated to compare the rate of endotracheal intubation across treatment arms. Number and percentage of subjects meeting and not meeting the primary endpoint will be tabulated per treatment arm. Frequency of missing data per treatment arm will be quantified. Ratios of relative risk will be presented with 95% confidence intervals. Relative risk estimate with 95% confidence intervals (Wald Normal approximation) will be calculated by log binomial regression with treatment as a predictor variable
- A superiority hypothesis test (two-sided, two-proportion Z test) will be carried out to investigate whether the rate of endotracheal intubation differs between intervention and standard-of-care. In this analysis, infants intubated for signs of respiratory failure (whether or not they strictly met the pre-defined criteria in the protocol) will be considered to have met the primary endpoint.

Secondary endpoints analysis

Mortality is an intercurrent event that could potentially occur before observation of many of the secondary endpoints in this trial in addition to the primary endpoint. Most secondary outcomes will involve evaluation of the intervention effect to be expected in infants while alive. Analysis of secondary endpoints will be performed on the intention-to-treat set of all randomised infants. The exceptions to this are

  - Number of attempts taken to successfully intubate in the delivery room – will only be assessed for those who need to be intubated in the delivery room (intubation in the delivery room is indicated by variable dr_infant_intub.factor)
  - Rectal temperature on admission to NICU as well as NICU intubation will only be evaluated for infants who are admitted to NICU alive (identified by derived variable nicu_admission)

- Bronchopulmonary dysplasia (BPD) – supplemental O_2 at 28 days of life - will only be evaluated for infants who are alive at 28 days of life (infants alive at 28 days to be determined by the variable bpd_d28.factor)
- Chronic lung disease of prematurity (CLD), the need for supplemental O_2 at 36 weeks corrected gestational age (CGA) determined by physiological oxygen reduction test - will only be evaluated for infants who are alive at 36 weeks CGA (determined by the variable cld_36wks.factor)
- Duration of hospitalisation will be summarized and analyzed both for infants who survive to hospital discharge and for the whole sample. A similar approach will be used for days of mechanical ventilation and days of respiratory support.

Each categorical secondary endpoint will be analysed using the same approach as the main analysis of the primary endpoint.

For the time-to-event endpoint death before hospital discharge, Kaplan-Meier curves will be constructed to visualize the pattern of mortality over the study period, with median survival time reported with 95% confidence intervals. Cox proportional hazards regression will be used to quantify the hazard ratio for those treated with oropharyngeal surfactant vs standard-of-care. A Cox proportional hazards regression model will also be fitted to determine sensitivity of the estimated intervention effect to potentially relevant covariates; including centre, gestational age, birth weight, gender, mode of delivery (vaginal versus caesarean birth) and antenatal corticosteroid treatment.

Each numeric secondary endpoint (e.g. duration of mechanical ventilation, duration of hospitalisation), will be analysed using the following approach.

- Descriptive statistics will be calculated to compare the endpoint across treatment groups. This will include the frequency of missing data per treatment arm, minimum and maximum values, mean and standard deviation for symmetrically distributed variables and median and interquartile range for asymmetrically distributed variables. The difference in means (or medians where relevant) between treatment groups will be presented with a 95% confidence interval. Confidence intervals for the difference in means will use a Normal approximation while confidence intervals for the difference in medians will be calculated by bootstrap resampling.
- A superiority hypothesis test (two-sided independent sample t-test) will be carried out to test for a difference in means between control and intervention. Alternatively, if t-test assumptions are not valid, a Mann-Whitney U test will be carried out to test for a difference in the the endpoint between control and intervention.

5.1.2 Sensitivity analyses

Sensitivity analysis will be conducted for the primary endpoint as follows.

- The sensitivity of the estimated intervention effect to measured covariates of interest (centre, gestational age, birth weight, gender, mode of delivery (vaginal versus caesarean birth) and antenatal corticosteroid treatment) will be evaluated by regression modelling. Generalized estimating equations will be fitted to the primary endpoint, with predictors including treatment and the above covariates. The relative risk of occurrence of the primary endpoint will be calculated for intervention vs placebo after adjustment

for the above covariates using binomial generalized estimating equations with the small sample Kauermann and Carroll correction oft he variance estimator used to calculate 95% confidence intervals around the relative risk.

- A competing risks model will be applied to investigate the effect of the intervention on the primary endpoint, adjusting for other outcomes that may impact on observation of the primary endpoint (e.g. death occurring before 120 hours of birth or intubation prior to 120 hours without meeting the criteria for respiratory failure defined in the protocol), should these competing risks occur.
- For infants intubated without meeting the pre-defined criteria for respiratory depression in the protocol, a sensitivity analysis will be conducted by assuming these infants would not have met the primary endpoint (i.e. as negative on the primary endpoint). Analysis will proceed as for the main analysis oft he primary endpoint.
- A per protocol analysis of the primary endpoint will be conducted to determine whether the treatment effect is sensitive to protocol deviations. Analysis will proceed as for the main analysis of the primary endpoint

5.1.3 Subgroup analyses

Subgroups of interest include infants of different gestational age strata (e.g. less than 26 weeks, and 26-28 weeks gestation at birth), and infants from different participating centres. Subgroup analysis of the primary endpoint and the important secondary outcome of death before hospital discharge will be carried out for these subgroups by:

- For each subgroup, the primary endpoint will be summarized per treatment arm by frequency and percentage, including the frequency of missing data. Relative risk of the primary endpoint will be estimated along with 95% confidence intervals for each subgroup, as per the main analysis of the primary endpoint
- Log binomial regression of the primary endpoint will be conducted to evaluate interaction effects for treatment type and centre (if centre sample size permits), and for treatment type and gestational age, with the acknowledgement that this analysis is not likely to have sufficient statistical power

5.2 Interim analyses

Interim analysis of the primary outcome and the important secondary outcome of death before hospital discharge will be performed on half of the planned sample (126) by a DSMB. The Haybittle-Peto boundary will be used as a stopping rule. Therefore, the DSMB may recommend terminating the study early for large and statistically significant differences ($p<0.001$) between groups in the primary outcome or in the important secondary outcome of death before hospital discharge; or for unanticipated concerns regarding the safety of enrolled infants.

5.2.1 Adjustment of Confidence intervals and p-values

The type I error rate for interim analysis will be set to 0.001 in accordance with the Haybittle-Peto stopping boundary. For final analysis, the type I error rate will be set at 0.05. Confidence intervals will be reported at a

95% confidence level.

5.3 Missing data

All data will be checked for missing values and followed-up to obtain data where possible. Missing data will be summarised by variable and by treatment arm. Spurious or anomalous data will also be queried and corrected if found to be incorrect. No data will be excluded as outliers.

For the handling of missing data, we will follow the flowchart given in [1]. If there is few (<5%) or a lot (>40%) of missing data, we will only use the observed data for the analysis.

With a low frequency of missing data (<5%), we will also perform worst case/best case sensitivity analysis for the study endpoints.

With missingness between 5 and 40% missing data will be replaced using multiple imputation. Multiple imputations will be based on the randomization arm, and selected baseline, procedural and outcome variables (see below for selection criteria). Since missing values in these variables are possible, chained equations will be used (e.g. using mi impute chained in Stata[2] or the mice[3] package in R[4]). This procedure fills in missing values in multiple variables iteratively based on a sequence of univariate imputation methods with fully conditional specification of prediction equations. Predictive mean matching will be used for continuous, logistic regression for binary, ordinal logistic regression for ordinal and multinomial regression for categorical variables (if applicable). Based on such chained equations, a total of 50 multiple imputations will be calculated. If it is not possible to impute all outcomes in one model, a stepwise approach will be considered. The 50 imputed data sets will be analysed using Rubin's rules[5].

Variables will be selected for inclusion in the imputation model using the following principles:

- Variables with more than 40% missing values will not be used
- Binary variables with a frequency of less than 5% in one category will not be used
- If two binary variables have less than 5% or more than 95% discordant pairs only one of the two will be used (the one with less missings).
- Levels of ordinal variables with a frequency of less than 5% will be collapsed by adding the entries to the neighbouring category (i.e. next higher or lower level) with the higher frequency (if there are more than one).
- Categorical variables might generally be recoded if it improves the fit of the imputation model and does not lead to substantial loss of information.
- Continuous variables will be log-transformed if it improves normality (checked by Shapiro-Wilk tests and QQ plots).

From the remaining variables an imputation model will be constructed based on combined clinical and statistical reasoning. All variables that may provide information about the imputed variable will be included.

5.4 Safety evaluation

Adverse events will be summarized for each site and for the whole sample, including:

- Total number of adverse events
  - Number and percentage of adverse events that were serious (SAES)
  - Number and percentage of adverse events that were mild, moderate and severe
  - Number and percentage of treatment-related adverse events
  - Number and percentage of adverse events that were resolved without sequalae, resolved with sequalae, ongoing, or resulted in death.
- Number of deaths (all causes) per treatment arm
- Number of deaths resulting from adverse events per treatment arm
- Number and percentage of subjects experiencing
  - Any adverse event
  - Any serious adverse event
  - Mild, moderate and severe adverse events
  - Treatment-related adverse events

Further, any non-serious adverse event occurring with a greater than 5% frequency in either treatment arm, will be described by treatment arm, including:

- System organ class
- Event term
- Number of subjects per treatment arm affected by the event
- Total number of events of this type that occurred per treatment arm

For all serious adverse events (SAEs), the above information will be reported as well as:

- Number of these SAEs causally related to treatment, per treatment arm
- Number of these SAEs with fatal outcome, per treatment arm
- Number of treatment-related SAEs of this type with fatal outcome

5.5 Statistical software

The statistical analysis will be performed using the statistical software packages Stata[2] and/or R[4].

5.6 Quality control

An independent statistician will conduct all analysis of the primary endpoint and key secondary endpoints. A statistician at UCD CRC will reproduce the primary of the primary endpoint based on the exported data as a quality control check. P-values of the primary analysis of the primary endpoint should be the same and limits of

the corresponding 95% confidence interval should not differ by more than 0.5 percentage points. Otherwise, the reason for the difference will be determined and a consensus must be reached.

6. Changes from the protocol

The SAP is consistent with the main features of the statistical methods described in the protocol.

7. References

[1] Jakobsen, J. C., Gluud, C., Wetterslev, J., & Winkel, P. (2017). When and how should multiple imputation be used for handling missing data in randomised clinical trials–a practical guide with flowcharts. BMC medical research methodology, 17(1), 1-10.

[2] StataCorp. 2021. Stata Statistical Software: Release 17. College Station, TX: StataCorp LLC

[3] van Buuren, S. & Groothuis-Oudshoorn, K. (2011). mice: Multivariate Imputation by Chained Equations in R. Journal of Statistical Software, 45 (3), 1-67

[4] R Development Core Team. 2008. R: A language and environment for statistical computing. R Foundation for Statistical Computing. Vienna, Austria

[5] Rubin, D. B. Multiple imputation for nonresponse in surveys. John Wiley & Sons, 2004, 81
