# Supplementary material for: Prophylactic Oropharyngeal Surfactant for Preterm Newborns at Birth: A Randomized Clinical Trial
Source: JAMA Pediatr. 2023 Dec 11;178(2):117–24. doi: 10.1001/jamapediatrics.2023.5082 (PMC10714282; doi:10.1001/jamapediatrics.2023.5082)
Supplement: Supplement 2. — eAppendix 1. Sensitivity Analysis eTable 1. Primary Analysis of the Primary End Point eTable 2. Sensitivity Analysis 1 of the Primary End Point eTable 3. Sensitivity Analysis 2 of the Primary End Point eTable 4. Summary of Competing Risks for the Primary End Point eFigure. Cumulative Incidence Functions for Competing Risks for the Primary End Point eTable 5. Competing Risk Analyses for the Primary End Point eTable 6. Logistic Generalized Estimating Equation (GEE) Fitted to the Primary End Point, Adjusting the Estimated Treatment Effect for Potentially Relevant Covariates eTable 7. Sensitivity Analysis 5 for the Primary End Point eTable 8. Sensitivity Analysis 6 for the Primary End Point eTable 9. Sensitivity Analysis for Secondary End Points Originally Analyzed by Complete-Case Analysis eAppendix 2. Subgroup Analysis eTable 10. Relative Risk of Primary End Point, Intubation for Respiratory Failure Within 120 Hours of Birth, for Intervention vs Control Reported by Category of Gestational Age eTable 11. Relative Risk of Primary End Point for Intervention vs Control, by Center eTable 12. Log Binomial Regression Evaluating Whether Treatment Effect on the Primary End Point Differs by Gestational Age Subgroup eAppendix 3. Adverse Events eTable 13. Total Number of Adverse Events, Serious Adverse Events (SAEs), Severity, Relatedness to Treatment and Outcome of Adverse Events Occurring by Treatment Arm and in Total During the POPART Trial eTable 14. Subjects With Adverse Events, by Treatment Group eTable 15. Complete Listing of Serious Adverse Events (SAEs) eTable 16. Listing of Nonserious Adverse Events eReference [file jamapediatr-e235082-s002.pdf]

Supplemental Online Content

Murphy MC, Miletin J, Klingenberg C, et al. Prophylactic oropharyngeal surfactant for preterm newborns at birth: a randomized clinical trial. *JAMA Pedatr*. Published online December 11, 2023. doi:10.1001/jamapediatrics.2023.5082

eAppendix 1. Sensitivity Analysis
eTable 1. Primary Analysis of the Primary End Point
eTable 2. Sensitivity Analysis 1 of the Primary End Point
eTable 3. Sensitivity Analysis 2 of the Primary End Point
eTable 4. Summary of Competing Risks for the Primary End Point
eFigure. Cumulative Incidence Functions for Competing Risks for the Primary End Point
eTable 5. Competing Risk Analyses for the Primary End Point
eTable 6. Logistic Generalized Estimating Equation (GEE) Fitted to the Primary End Point, Adjusting the Estimated Treatment Effect for Potentially Relevant Covariates
eTable 7. Sensitivity Analysis 5 for the Primary End Point
eTable 8. Sensitivity Analysis 6 for the Primary End Point
eTable 9. Sensitivity Analysis for Secondary End Points Originally Analyzed by Complete-Case Analysis
eAppendix 2. Subgroup Analysis
eTable 10. Relative Risk of Primary End Point, Intubation for Respiratory Failure Within 120 Hours of Birth, for Intervention vs Control Reported by Category of Gestational Age
eTable 11. Relative Risk of Primary End Point for Intervention vs Control, by Center
eTable 12. Log Binomial Regression Evaluating Whether Treatment Effect on the Primary End Point Differs by Gestational Age Subgroup
eAppendix 3. Adverse Events
eTable 13. Total Number of Adverse Events, Serious Adverse Events (SAEs), Severity, Relatedness to Treatment and Outcome of Adverse Events Occurring by Treatment Arm and in Total During the POPART Trial
eTable 14. Subjects With Adverse Events, by Treatment Group
eTable 15. Complete Listing of Serious Adverse Events (SAEs)
eTable 16. Listing of Nonserious Adverse Events
eReference

This supplemental material has been provided by the authors to give readers additional information about their work.

© 2023 Murphy MC et al. *JAMA Pediatrics.*

eAppendix 1. Sensitivity Analysis

Primary end point analysis

The primary end point is endotracheal intubation for respiratory failure within 120 hours of birth. Enrolled infants were intubated for persistent apnea and/or bradycardia in the DR, or for respiratory failure in the NICU defined as ≥ 2 of:

• Clinical signs – worsening tachypnoea; grunting; subcostal, intercostal and/or sternal recession

• Acidosis – pH < 7.2 on 2 blood gases (arterial or capillary) ≥ 30 minutes apart

• O2 –FiO2 >0.4 to keep SpO2 ≥90% for >30 minutes • PCO2 > 9.0 kPa on 2 blood gases (arterial or capillary) ≥ 30 minutes apart

• Apnea – recurrent apnea treated with mask ventilation The frequency of blood gas monitoring is based on the clinical decision of the treating physician, as per routine practice.

© 2023 Murphy MC et al. *JAMA Pediatrics.*

Primary analysis of the primary end point

Table (eTable 1) below shows the results of intention-to-treat analysis of the primary end point. The main analysis of the primary end point is conducted on the Full Analysis Set (FAS), utilizes data on subjects up to 120 hours of life, while alive, and assumes that infants intubated within 120 hours met the protocol defined criteria for respiratory failure. There was no difference in the primary end point between groups (RR 0.98, 95% CI:0.81-1.18; Z=0.08, p=0.93).

eTable 1. Primary Analysis of the Primary End Point

**End Point**		**Oropharyngeal surfactant (N=126)**	**No intervention (N=125)**	**RR for intervention vs control (95% CI)**	**Z test statistic (p-value)**
Intubation for respiratory failure within 120 hours of birth	Yes	80 (63.5%)	81 (64.8%)	0.9798 (0.8143-1.1789)	0.084 (0.933)
	No	46 (36.5%)	44 (35.2%)		
	N-Miss	0	0		

Sensitivity analysis of the primary end point

There were a number of pre-defined sensitivity analysis for the primary end point defined in the protocol.

- Sensitivity analysis 1 – per protocol analysis of the primary end point, excluding subjects with major protocol deviations.
- Sensitivity analysis 2 – assumes that infants who were intubated without strictly meeting the protocol defined criteria for respiratory failure (listed above) did not meet the primary end point.

© 2023 Murphy MC et al. *JAMA Pediatrics.*

- Sensitivity analysis 3 – competing risks analysis of primary end point, accounting for competing risks of death before intubation within 120 hours of birth, or intubation within 120hrs without meeting the specific criteria defined in the protocol. A competing risks model was fitted using the cmprsk package in R (https://CRAN.R-project.org/package=cmprsk) to investigate the effect of the intervention on the primary end point, adjusting for competing outcomes (e.g. mortality) that may impact on observation of the primary end point. Gray's test[1] was used to test for a difference between treatment arms in the fitted cumulative incidence functions (CIFs).

- Sensitivity analysis 4 – Covariate-adjusted analysis of the primary end point. The sensitivity of the estimated intervention effect to measured covariates of interest, including center, GA, BW, gender, mode of delivery and antenatal corticosteroid treatment, were evaluated with regression analysis.

Sensitivity analyses 1-4 of the primary end point were prespecified in the protocol. We have added here two additional sensitivity analyses. Sensitivity analysis 5 considers the impact of omission of one infant from the analysis set. This infant was diagnosed with esophageal atresia after birth and was excluded from study after diagnosis and a decision was made to exclude this subject from the Full Analysis Set. Sensitivity analysis 5 was conducted to ensure primary end point analysis was not substantially impacted by this decision.

Sensitivity analysis 6 was conducted to determine whether the estimated treatment effect was impacted by the decision to analyze all infants as independent, not accounting for the fact that infants born in the same gestation were enrolled and randomized as individuals. To test the impact of this assumption on the estimated treatment effect, sensitivity analysis 6 accounts for the fact that outcomes of infants of the same gestation can be correlation. A generalized estimating equation (GEE) model was fitted to the primary end point, intubation for respiratory failure within 120 hours

© 2023 Murphy MC et al. *JAMA Pediatrics.*

of birth, to estimate the effect of treatment on this end point. The model is fitted with an assumed exchangeable correlation structure i.e. assuming that outcomes observed from babies from the same gestation (within each mother) are equally correlated.

Therefore, two additional sensitivity analysis were conducted that were not pre-specified in the trial protocol:

Sensitivity Analysis 5: includes all infants in the groups to which they were randomized, regardless of post-randomization exclusion from the trial.

Sensitivity Analysis 6: log binomial generalized estimating equation (GEE) to estimate the effect of the intervention on the primary end point, accounting for the fact that outcomes from infants of the same gestation may be correlated.

The following subsections show the results of all sensitivity analysis on the primary end point. Results of sensitivity analysis do not disagree with the results of primary analysis of the primary end point.

Sensitivity analysis 1 for primary end point

This is a per protocol analysis of the primary end point (eTable 2). It excludes two subjects who were included in the intention-to-treat set who did not meet trial eligibility criteria. Conclusions agree with the primary analysis of the primary end point, with similar relative risk.

eTable 2. Sensitivity Analysis 1 of the Primary End Point

**End Point**		**Oropharyngeal surfactant (N=126)**	**No intervention (N=123)**	**RR for intervention vs control (95% CI)**	**Z test statistic (p-value)**
Intubation within 120 hours of birth	Yes	80 (63.5%)	79 (64.2%)	0.989 (0.82-1.192)	0.084 (0.933)
	No	46 (36.5%)	44 (35.8%)		
	N-Miss	0	0		

© 2023 Murphy MC et al. *JAMA Pediatrics.*

Sensitivity analysis 2 for primary end point

This sensitivity analysis (eTable 3) assumes that infants who were intubated without strictly meeting the protocol defined criteria would not have met the primary end point. Conclusions agree with the primary analysis of the primary end point, with similar relative risk.

eTable 3. Sensitivity Analysis 2 of the Primary End Point

**End Points**		**Oropharyngeal Surfactant (N=126)**	**No intervention (N=125)**	**RR for intervention vs control (95% CI)**	**Z test statistic (p-value)**
Infants intubated without meeting the pre-defined criteria for RDS	Yes	69 (54.8%)	72 (57.6%)	0.951 (0.762, 1.184)	0.326 (0.745)
	No	57 (45.2%)	53 (42.4%)		
	N-Miss	0	0		

Sensitivity analysis 3 for primary end point

This sensitivity analysis (eTable 4) accounts for competing risks in estimation of treatment effect on the primary end point. Competing risks for observation of the primary end point could include death before intubation within 120 hours of birth, or intubation within 120hrs without meeting the specific criteria defined in the protocol. In the trial, any infants who died within 120hrs met the primary end point first, and so the analysis does not need to account for death as a competing risk. To evaluate sensitivity of results to the reason for intubation (meeting pre- defined criteria or not), a competing risks analysis was carried out using the cmprsk package in R.

© 2023 Murphy MC et al. *JAMA Pediatrics.*

eTable 4. Summary of Competing Risks for the Primary End Point

Reason for first intubation (within 120hrs of life)	Oropharyngeal Surfactant	No Intervention	Total
Not intubated within 120 hours	46 (36.5%)	44 (35.2%)	90 (35.9%)
Intubated within 120 hours before meeting pre-defined criteria	11 (8.7%)	9 (7.2%)	20 (8.0%)
Intubated within 120 hours for meeting pre-defined criteria	69 (54.8%)	72 (57.6%)	141 (56.2%)
Total	126 (100.0%)	125 (100.0%)	251 (100.0%)

© 2023 Murphy MC et al. *JAMA Pediatrics.*

eFigure. Cumulative Incidence Functions for Competing Risks for the Primary End Point

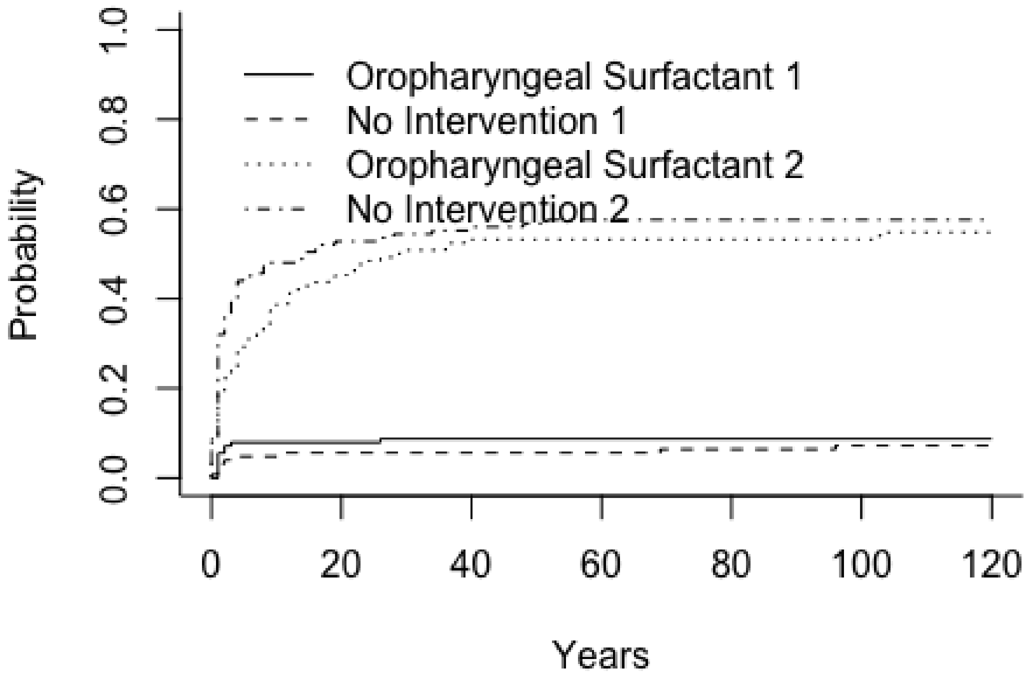

The figure above (eFigure 1) depicts cumulative incidence curves for intubation within 120 hours, by reason and by treatment group, estimated by completing risks analysis. The cumulative incidence function (CIF) shows the probability of an infant experiencing intubation by a specified hour of life (x-axis) in the presence of competing risks. Numerical estimates of the CIF function at 120 hours of life are shown in the below table (eTable 5), along with Gray's test comparing CIF functions between treatment groups for each cause of intubation (meeting pre-defined PE criteria and not). The between-group difference in CIF functions is not statistically significant for either event (intubation meeting primary end point criteria and not meeting primary end point criteria defined in the protocol). Results are therefore not contradictory of results of the primary analysis of the primary end point.

© 2023 Murphy MC et al. *JAMA Pediatrics.*

eTable 5. Competing Risk Analyses for the Primary End Point

Event	CIF	95% Confidence interval Lower limit	Upper limit	Gray's test
Oropharyngeal Surfactant - PE criteria not met	0.087	0.046	0.145	χ2 = 0.208, df=1, p=0.648
No Intervention - PE criteria not met	0.072	0.035	0.126	
Oropharyngeal Surfactant - PE criteria met	0.548	0.457	0.63	χ2 = 0.778, df=1, p=0.378
No Intervention - PE criteria met	0.576	0.485	0.657	

CIF – cumulative incidence function

Sensitivity analysis 4 for primary end point

Table (eTable 6) below shows the results of covariate-adjusted analysis of the primary end point. A generalized estimating equation (GEE) model was fitted to the primary end point, intubation for respiratory failure within 120 hours of birth, to estimate the effect of treatment on this end point while adjusting for relevant covariates, including gestational age category, center, gender, doses of antenatal steroids, mode of delivery and birth weight. Within center correlation was allowed for using an exchangeable correlation structure.

Note the statistical analysis plan specified a log binomial regression for this data to estimate relative risk and confidence intervals, however, model fitting issues due to out of range probabilities made this infeasible, and hence the model was fitted with a logit link, giving estimates for Odds Ratio and corresponding 95% confidence interval in the table below.

The odds ratio is close to one, and p-value also close to one, indicating negligible difference in the odds of the primary end point for the intervention vs control arms, once covariates are adjusted for. Hence, results agree with the main analysis of the primary end point.

© 2023 Murphy MC et al. *JAMA Pediatrics.*

eTable 6. Logistic Generalized Estimating Equation (GEE) Fitted to the Primary End Point, Adjusting the Estimated Treatment Effect for Potentially Relevant Covariates

						95% Confidence interval for OR	
**Variable**	**Estimate**	**SE**	**Z**	**p-value**	**OR**	**Lower limit**	**Upper limit**
(Intercept)	3.501	1.034	11.464	0.001	33.158	4.369	251.655
**Treatment group (Oropharyngeal Surfactant)**	**-0.020**	**0.209**	**0.009**	**0.923**	**0.980**	**0.651**	**1.476**
Gestational age (26-28w)	-0.567	0.214	7.033	0.008	0.567	0.373	0.862
Gender (Female)	-0.111	0.337	0.108	0.742	0.895	0.463	1.732
Caesarean delivery	0.349	0.167	4.375	0.036	1.418	1.022	1.967
Doses of antenatal steroids	-0.288	0.088	10.658	0.001	0.750	0.631	0.891
Birth weight	-0.002	0.001	15.325	0.000	0.998	0.996	0.999

Sensitivity analysis 5 for primary end point

Table (eTable 7) shows the results of sensitivity analysis 5 on the primary end point. One infant (randomized to the intervention arm) was removed from the study and subsequently from the Full Analysis Set for meeting study exclusion criteria. No follow-up data on this infant was available. To determine whether this omission from the Full Analysis Set had an impact on primary end point results, sensitivity analysis is conducted here for the primary end point analysis, with this additional infant included. The main analysis is repeated with this additional subject, under two conditions (i) assuming this infant met the primary end point and (ii) assuming this infant did not meet the primary end point. No material difference in results is observed.

© 2023 Murphy MC et al. *JAMA Pediatrics.*

Sensitivity analysis 5 for the primary end point

eTable 7. Sensitivity Analysis 5 for the Primary End Point

	**Intubation for respiratory failure within 120 hours**			
	**No**	**Yes**	**RR (95% CI)**	**Z test**
**Main analysis of the PE (n=251)**				
No Intervention	44 (35.2%)	81 (64.8%)		
Oropharyngeal Surfactant	46 (36.5%)	80 (63.5%)	0.978 (0.814-1.179)	Z = 0.08, p = 0.93
**Assuming omitted infant met the primary end point (n=252)**				
No Intervention	44 (64.8%)	81 (35.2%)		
Oropharyngeal Surfactant	46 (63.8%)	81 (36.2%)	1.029 (0.739-1.433)	Z = 0.04, p = 0.97
**Assuming omitted infant did not meet the primary end point (n=252)**				
No Intervention	44 (64.8%)	81 (35.2%)		
Oropharyngeal Surfactant	47 (63%)	80 (37%)	1.051 (0.757-1.461)	Z = 0.17, p = 0.87

Sensitivity analysis 6 for the primary end point

The 251 infants included in intention-to-treat analysis were born to 208 mothers. There were 171 mothers with one infant enrolled, 31 mothers with two infants enrolled and 6 mothers with three infants enrolled. eTable 8 shows the results of sensitivity analysis of the primary end point, whereby correlation between infants of the same gestation is accounted for during modelling. A generalized estimating equation (GEE) model was fitted to the primary end point, intubation for respiratory failure within 120 hours of birth, to estimate

© 2023 Murphy MC et al. *JAMA Pediatrics.*

the effect of treatment on this end point. The model is fitted with an assumed exchangeable correlation structure i.e. assuming that outcomes observed from babies from the same gestation (within each mother) are equally correlated. Shown in the table are the estimated relative risk with 95% confidence intervals (formed from robust standard errors) and the results of Wald test on the regression coefficient for treatment (intervention vs control). The estimates do not deviate substantially from those of the main primary end point analysis.

eTable 8. Sensitivity Analysis 6 for the Primary End Point

Arm	No	Yes	RR	Z.test
No Intervention	44 (35.2%)	81 (64.8%)		
Oropharyngeal Surfactant	46 (36.5%)	80 (63.5%)	0.995 (0.829-1.195)	Z=0.003, p=0.96

Sensitivity analysis for secondary end points

Sensitivity analysis was conducted for secondary end points that were analyzed by complete case analysis for the main analysis, in accordance with the statistical analysis plan for the study. For these secondary end points, the main analysis was repeated twice (using the same analysis approach) for end points with less than 5% missingness per treatment arm, using best-worst and worst-best case imputation. For example, the end point BPD at 28 days of life had 2 missing values in the intervention arm and 1 in the control arm. In best-worst case imputation, subjects in the intervention arm with missing data are assumed to have had BPD at 28 days while subjects in the control arm with missing data are assumed not to have had BPD at 28 days. In worst-best case imputation, subjects in the intervention arm are assumed not to have had BPD at 28 days while subjects in the control arm are assumed to have had BPD at 28 days. The same analysis approach as was used for the main analysis is then applied.

© 2023 Murphy MC et al. *JAMA Pediatrics.*

Results of sensitivity analysis for each relevant secondary end point are presented below the results of main analysis for that end point (eTable 9). Results of sensitivity analysis do not differ substantially from results of the main analysis of secondary end points.

eTable 9. Sensitivity Analysis for Secondary End Points Originally Analyzed by Complete-Case Analysis

End points	Missing data handling		Oropharyngeal surfactant (N=126)	No intervention (N=125)	Relative Risk/Difference in Median for Oropharyngeal surfactant vs No intervention	Test statistic (p-value)
Number of attempts to delivery room intubation [1]	Complete Case Analysis	Median (Q1, Q3)	1.0 (1.0, 2.0)	1.0 (1.0, 2.0)	0.0 (-0.761, 1.243)	0.849 (0.396)
		Range	1.0 - 5.0	1.0 - 6.0		
		N-miss	0[R1]	1[R1]		
	Best-worst Case Analysis	Median (Q1, Q3)	1.0 (1.0, 2.0)	1.0 (1.0, 2.0)	0.0 (-0.674, 1.298)	1.030 (0.303)
	Worst-best Case Analysis	Median (Q1, Q3)	1.0 (1.0, 2.0)	1.0 (1.0, 2.0)	0.0 (-0.718, 1.112)	0.760 (0.447)
Number of days on respiratory support [2]	Complete Case Analysis	Median (Q1, Q3)	53 (27.2, 73.0)	50 (25.5, 70.2)	3.0 (-5.472, 14.728)	0.218 (0.827)
		Range	0.0 - 321.0	0.0 - 451.0		
		N-miss	0	1		
	Best-worst Case Analysis	Median (Q1, Q3)	53 (27.2, 73.0)	50 (25.5, 70.2)	3.0 (-5.330, 15.147)	0.326 (0.744)
		Median (Q1, Q3)	53 (27.2, 73.0)	50 (25.5, 70.2)	3.0 (-5.739, 14.537)	0.108 (0.914)

© 2023 Murphy MC et al. *JAMA Pediatrics.*

	Worst-best Case Analysis					
Bronchopulmonary dysplasia (BPD) supplemental oxygen at 28 days of life [3]	Complete Case Analysis	Yes	73 (69.52%)	74 (69.16%)	1.005 (0.838, 1.206)	0.000 (1.000)
		No	32 (30.48%)	33 (30.84%)		
		N-Miss	2[R3]	1[R3]		
	Best-worst Case Analysis	Yes	73 (68.22%)	75 (69.44%)	0.982 (0.818, 1.179)	0.046 (0.963)
		No	34 (31.78%)	33 (30.56%)		
	Worst-best Case Analysis	Yes	75 (70.09%)	74 (68.52%)	1.023 (0.854, 1.227)	0.103 (0.918)
		No	32 (29.91%)	34 (31.48%)		
Chronic lung disease of prematurity (CLD) need for supplemental oxygen at 36 weeks [4]	Complete Case Analysis	Yes	28 (27.18%)	30 (29.41%)	0.924 (0.598, 1.426)	0.19 (0.84)
		No	75 (72.82%)	72 (70.59%)		
		N-Miss	0[R4]	1 [R4]		
	Best-worst Case Analysis	Yes	28 (27.18%)	31 (30.10%)	0.903 (0.587, 1.387)	0.308 (0.758)
		No	75 (72.82%)	72 (69.90)		
	Worst-best Case Analysis	Yes	28 (27.18%)	30 (29.13%)	0.933 (0.604, 1.440)	0.155 (0.877)
		No	75 (72.82%)	73 (70.87%)		
Surgical treatment of patent ductus arteriosus	Complete Case Analysis	Yes	2 (1.6%)	2 (1.6%)	0.992 (0.177, 5.556)	0.000 (1.000)
		No	122 (98.4%)	121 (98.4%)		
		N-Miss	2	2		

© 2023 Murphy MC et al. *JAMA Pediatrics.*

	Best-worst Case Analysis	Yes	2 (1.6%)	4 (3.2%)	0.496 (0.108, 2.275)	0.179 (0.672)
		No	124(98.4%)	121 (96.8%)		
	Worst-best Case Analysis	Yes	4(3.2%)	2 (1.6%)	1.984 (0.433, 9.156)	0.163 (0.687)
		No	122 (96.8%)	123 (98.4%)		
Incidence of intraventricular hemorrhage	Complete Case Analysis	Yes	16 (13.2%)	18 (14.5%)	0.911 (0.492, 1.685)	0.012 (0.914)
		No	105 (86.8%)	106 (85.5%)		
		N-Miss	5	1		
	Best-worst Case Analysis	Yes	16 (12.7%)	19 (15.2%)	0.835 (0.454, 1.533)	0.152 (0.697)
		No	110 (87.3%)	106 (84.8%)		
	Worst-best Case Analysis	Yes	21 (16.7%)	18 (14.4%)	1.157 (0.654, 2.053)	0.103 (0.748)
		No	105 (83.3%)	107 (85.6%)		
Incidence of severe intraventricular hemorrhage	Complete Case Analysis	Yes	7 (5.8%)	9 (7.3%)	0.797 (0.316, 2.002)	0.043 (0.835)
		No	114 (94.2%)	115 (92.7%)		
		N-Miss	5	1		
	Best-worst Case Analysis	Yes	7 (5.6%)	10 (8.0%)	0.694 (0.249, 1.926)	0.270 (0.604)
		No	119 (94.4%)	115 (92.0%)		

© 2023 Murphy MC et al. *JAMA Pediatrics.*

	Worst-best Case Analysis	Yes	12 (9.5%)	9 (7.2%)	1.323 (0.592, 2.969)	0.191 (0.662)
		No	114 (90.5%)	116 (92.8%)		
Incidence of cystic periventricular leukomalacia	Complete Case Analysis	Yes	4 (3.3%)	5 (4.0%)	0.820 (0.243, 2.759)	0.000 (1.000)
		No	117 (96.7%)	119 (96.0%)		
		N-Miss	5	1		
	Best-worst Case Analysis	Yes	4 (3.2%)	6 (4.8%)	0.661 (0.204, 2.131)	0.113 (0.737)
		No	122 (96.8%)	119 (95.2%)		
	Worst-best Case Analysis	Yes	9 (7.1%)	5 (4.0%)	1.786 (0.646, 4.969)	0.656 (0.418)
		No	117 (92.9%)	120 (96.0%)		
Survival without CLD at hospital discharge	Complete Case Analysis	Yes	71 (56.3%)	72 (58.1%)	0.971 (0.782, 1.204)	0.021 (0.884)
		No	55 (43.7%)	52 (41.9%)		
		N-Miss	0	1		
	Best-worst Case Analysis	Yes	71 (56.3%)	73 (58.4)	0.965 (0.778, 1.196)	0.201 (0.841)
		No	55 (43.7%)	52 (41.6%)		
	Worst-worst Case Analysis	Yes	71 (56.3%)	72 (57.6%)	0.978 (0.788, 1.215)	0.073 (0.942)
		No	55 (43.7%)	53 (42.4%)		
Use of home oxygen therapy		Yes	5 (4.0%)	10 (8.2%)	0.484 (0.177, 1.313)	1.130 (0.258)

© 2023 Murphy MC et al. *JAMA Pediatrics.*

	Complete Case Analysis	No	121 (96.0%)	112 (91.8%)		
		N-Miss	0	3		
	Best-worst Case Analysis	Yes	5 (4.0%)	13 (10.4%)	0.382 (0.145, 0.993)	1.730 (0.084)
		No	121 (96.0%)	112 (89.6%)		
	Worst-best Case Analysis	Yes	5 (4.0%)	10 (8.0%)	0.496 (0.181, 1.345)	1.081 (0.280)
		No	121 (96.0%)	115 (92.0%)		

[R1] Analyzed for infants intubated in the delivery room only (28 in the intervention arm and 38 in the control arm)
[R2] Defined as endotracheal ventilation, high-frequency oscillatory ventilation, CPAP, heated humidified high-flow nasal cannula O_2, low flow nasal cannula O_2
[R3] Applicable only to infants alive at 28 days of life (107 in the intervention arm and 108 in the control arm)
[R4] Applicable only to infants alive at 36 weeks post randomization (103 in the intervention arm and 103 in the control arm)

© 2023 Murphy MC et al. *JAMA Pediatrics.*

eAppendix 2. Subgroup Analysis

Subgroup analysis is performed by gestational age (<26 weeks and 26-28 weeks best estimate of gestational age) and by study center. Subgroup analysis is performed for the primary end point of intubation within 120 hours of birth and the secondary end point of death before hospital discharge. For each subgroup of interest, frequency table and relative risk (with 95% confidence interval) of the end point is calculated to evaluate effect of the intervention by subgroup, using the epitools package in R.

Due to smaller numbers recruited at some European centers, centers were re-categorized into four for calculation of relative risk, as NMH, Ireland; Coombe, Ireland; Norwegian centers (Bergen and Tromsø) and Other European centers. Log binomial regression models were fitted to each end point, including treatment group, gestational age category, and the interaction between gestational age and treatment group as model predictors. The interaction effect in these models can be used to evaluate whether treatment effect differed by gestational age category. Due to smaller sample sizes in center subgroups, the interaction with center was not explored. Log binomial regression models were fitted using the logbin package in R.

Note that this subgroup analysis reported here is exploratory by nature as sample size is likely not large enough for adequately powered subgroup analysis.

Subgroup analysis of the primary end point

Table (eTable 10) below shows relative risk of the primary end point by gestational age category for oropharyngeal surfactant vs standard-of-care (CPAP). In infants aged <26 weeks, incidence of the primary end point was higher for infants assigned to the intervention (85.4%) than for those assigned to no intervention (79.5%). However, the inverse is true for infants born at 26-28 weeks' gestation - incidence of the primary end point was lower in those assigned to the intervention (50%)

© 2023 Murphy MC et al. *JAMA Pediatrics.*

than those assigned to standard-of-care (56.8%). However, the difference would not be considered statistically significant, with relative risk confidence intervals overlapping with one for both subgroups, and a non-significant interaction term in the log binomial regression model (eTable 12).

Relative risk of the primary end point differs somewhat by center (eTable 11), whereby incidence of the primary end point was higher in the intervention group than control group in the two Norwegian centers combined, but lower for the intervention group than the control group in both Irish centers and the Other European centers (most markedly in the Coombe, Ireland). However, this is likely explained by relative small within-center sample sizes, and the treatment effect would not be considered statistically significant in any of these subgroups (confidence intervals for RR overlapping with 1).

eTable 10. Relative Risk of Primary End Point, Intubation for Respiratory Failure Within 120 Hours of Birth, for Intervention vs Control Reported by Category of Gestational Age

		Intubation for respiratory failure within 120 hours of birth		
Gestational age	Arm	No	Yes	RR (95% CI)
<26 weeks	No Intervention	9 (20.5%)	35 (79.5%)	
	Oropharyngeal Surfactant	7 (14.6%)	41 (85.4%)	1.074 (0.888-1.299)
26-28 weeks	No Intervention	35 (43.2%)	46 (56.8%)	
	Oropharyngeal Surfactant	39 (50%)	39 (50%)	0.880 (0.657-1.179)

© 2023 Murphy MC et al. *JAMA Pediatrics.*

eTable 11. Relative Risk of Primary End Point for Intervention vs Control, by Center

		Intubation for respiratory failure within 120 hours of birth		
Center	Arm	No	Yes	RR (95% CI)
NMH, Ireland	No Intervention	29 (43.9%)	37 (56.1%)	
	Oropharyngeal Surfactant	30 (46.2%)	35 (53.8%)	0.961 (0.704-1.310)
Coombe, Ireland	No Intervention	5 (20%)	20 (80%)	
	Oropharyngeal Surfactant	8 (34.8%)	15 (65.2%)	0.815 (0.570-1.165)
Norway	No Intervention	6 (33.3%)	12 (66.7%)	
	Oropharyngeal Surfactant	3 (14.3%)	18 (85.7%)	1.286 (0.888-1.862)
Other	No Intervention	4 (25%)	12 (75%)	
	Oropharyngeal Surfactant	5 (29.4%)	12 (70.6%)	0.941 (0.62-1.429)

eTable 12. Log Binomial Regression Evaluating Whether Treatment Effect on the Primary End Point Differs by Gestational Age Subgroup

Coefficient	Estimate	Std. Error	z value	p-value
(Intercept)	-0.229	0.076	-2.993	0.003
Treatment group (Oropharyngeal Surfactant)	0.071	0.097	0.734	0.463
Gestational age (26-28w)	-0.337	0.123	-2.73	0.006
Treatment group*Gestational age	-0.199	0.178	-1.117	0.264

Coefficient	Estimate	Std. Error	z value	p-value
(Intercept)	-0.229	0.076	-2.993	0.003
Treatment group (Oropharyngeal Surfactant)	0.071	0.097	0.734	0.463
Gestational age (26-28w)	-0.337	0.123	-2.73	0.006
Treatment group*Gestational age	-0.199	0.178	-1.117	0.264

© 2023 Murphy MC et al. *JAMA Pediatrics.*

eAppendix 3. Adverse Events

This section contains a summary of the adverse events that occurred during the POPART trial.

Adverse events by treatment arm

eTable 13. Total Number of Adverse Events, Serious Adverse Events (SAEs), Severity, Relatedness to Treatment and Outcome of Adverse Events Occurring by Treatment Arm and in Total During the POPART Trial

Adverse events summary	Oropharyngeal Surfactant	No Intervention	Total
Adverse events	39	35	74
SAEs	26 (66.7%)	24 (68.6%)	50 (67.6%)
Severity			
Mild	1 (2.6%)	2 (5.7%)	3 (4.1%)
Moderate	10 (25.6%)	8 (22.9%)	18 (24.3%)
Severe	28 (71.8%)	25 (71.4%)	53 (71.6%)
Relatedness to treatment			
Related	0 (0.0%)	0 (0.0%)	0 (0.0%)
Not Related	39 (100.0%)	35 (100.0%)	74 (100.0%)
Action taken			
None	16 (41.0%)	3 (8.6%)	19 (25.7%)
Drug Withdrawn/Stopped	0 (0.0%)	0 (0.0%)	0 (0.0%)
Drug Interrupted	0 (0.0%)	0 (0.0%)	0 (0.0%)
Not Applicable	23 (59.0%)	32 (91.4%)	55 (74.3%)
Outcome			

© 2023 Murphy MC et al. *JAMA Pediatrics.*

Adverse events summary	Oropharyngeal Surfactant	No Intervention	Total
Ongoing	3 (7.7%)	3 (8.6%)	6 (8.1%)
Resolved without Sequelae	11 (28.2%)	10 (28.6%)	21 (28.4%)
Resolved with Sequelae	3 (7.7%)	0 (0.0%)	3 (4.1%)
Death/Fatal	22 (56.4%)	22 (62.9%)	44 (59.5%)

© 2023 Murphy MC et al. *JAMA Pediatrics.*

Adverse events by subject

Table (eTable 14) below shows the total number of subjects with adverse events, SAEs, mild, moderate and severe AEs, number of subjects with treatment related AEs, number of subjects experiencing various AE outcomes, as well as median and interquartile range of the number of adverse events and serious adverse events per subject over the course of the POPART trial.

eTable 14. Subjects With Adverse Events, by Treatment Group

Measure	Oropharyngeal Surfactant (N=127)	No Intervention (N=125)	Total (N=252)
Subjects with AEs	30 (23.6%)	29 (23.2%)	59 (23.4%)
Subjects with SAEs	25 (19.7%)	23 (18.4%)	48 (19.0%)
Subjects with adverse events by severity			
Mild	1 (0.8%)	2 (1.6%)	3 (1.2%)
Moderate	6 (4.7%)	5 (4.0%)	11 (4.4%)
Severe	26 (20.5%)	23 (18.4%)	49 (19.4%)
Subjects with treatment-related adverse events	0 (0.0%)	0 (0.0%)	0 (0.0%)
Number of adverse events per subject			
Median (Q1, Q3)	0.0 (0.0, 0.0)	0.0 (0.0, 0.0)	0.0 (0.0, 0.0)
Range	0.0 - 4.0	0.0 - 5.0	0.0 - 5.0
Number of serious adverse events per subject			
Median (Q1, Q3)	0.0 (0.0, 0.0)	0.0 (0.0, 0.0)	0.0 (0.0, 0.0)
Range	0.0 - 2.0	0.0 - 2.0	0.0 - 2.0

© 2023 Murphy MC et al. *JAMA Pediatrics.*

Listing of Serious Adverse Events (SAEs)

Table (eTable 15) below contains a complete listing of serious adverse events (SAEs) for the POPART trial. Number of SAEs is reported by MedDRA preferred term (PT) and system organ class (SOC).

eTable 15. Complete Listing of Serious Adverse Events (SAEs)

System organ class	Event	Oropharyngeal surfactant (n=126)	No intervention (n=125)
10007541 Cardiac disorders	10007618 Cardio-respiratory arrest neonatal		1
10007541 Cardiac disorders	10037450 Pulmonary valve stenosis	1	
10010331 Congenital, familial and genetic disorders	10037407 Pulmonary hypoplasia	2	
10015919 Eye disorders	10038923 Retinopathy	1	
10017947 Gastrointestinal disorders	10051606 Necrotizing colitis	1	3
10017947 Gastrointestinal disorders	10055667 Necrotizing enterocolitis neonatal	1	
10017947 Gastrointestinal disorders	10074160 Neonatal intestinal perforation	1	3
10018065 General disorders and administration site conditions	10011906 Death		2
10018065 General disorders and administration site conditions	10050401 Neonatal multi-organ failure	1	2
10021881 Infections and infestations	10035699 Pneumonia Escherichia	1	
10021881 Infections and infestations	10040049 Sepsis neonatal	3	
10021881 Infections and infestations	10053166 Candida sepsis		1
10021881 Infections and infestations	10053840 Bacterial sepsis	1	1
10029205 Nervous system disorders	10008111 Cerebral hemorrhage	1	
10029205 Nervous system disorders	10018985 Hemorrhage intracranial	1	2
10029205 Nervous system disorders	10052594 Periventricular leukomalacia	1	
10029205 Nervous system disorders	10070511 Hypoxic-ischemic encephalopathy		1

© 2023 Murphy MC et al. *JAMA Pediatrics.*

10036585 Pregnancy, puerperium and perinatal conditions	10036590 Premature baby	4	1
10038738 Respiratory, thoracic and mediastinal disorders	10006475 Bronchopulmonary dysplasia		1
10038738 Respiratory, thoracic and mediastinal disorders	10028974 Neonatal respiratory distress syndrome		2
10038738 Respiratory, thoracic and mediastinal disorders	10037394 Pulmonary hemorrhage		1
10038738 Respiratory, thoracic and mediastinal disorders	10038695 Respiratory failure	6	2
10038738 Respiratory, thoracic and mediastinal disorders	10082194 Pulmonary hemorrhage neonatal		1

© 2023 Murphy MC et al. *JAMA Pediatrics.*

Listing of non-serious adverse events

Finally, table (eTable 16) below lists all recorded non-serious adverse events in the POPART trial, alongside information on treatment arm allocation, severity, relatedness-to-treatment and outcome.

eTable 16. Listing of Nonserious Adverse Events

Event	Allocation	Severity	Related to treatment	Outcome
Congenital hypothyroidy	No Intervention	Moderate	Not Related	Ongoing
feeding disorder (need of NG tube)	No Intervention	Moderate	Not Related	Ongoing
GBS infection	Oropharyngeal Surfactant	Moderate	Not Related	Resolved without Sequelae
inguinal hernia operation	Oropharyngeal Surfactant	Moderate	Not Related	Resolved without Sequelae
IVH of degree III	Oropharyngeal Surfactant	Moderate	Not Related	Ongoing
Kidney Failure	Oropharyngeal Surfactant	Mild	Not Related	Resolved without Sequelae
Lactobezoar	No Intervention	Moderate	Not Related	Resolved without Sequelae
LACTOBEZOAR	Oropharyngeal Surfactant	Moderate	Not Related	Resolved without Sequelae
Necrotizing enterocolitis	Oropharyngeal Surfactant	Severe	Not Related	Resolved without Sequelae
PDA	No Intervention	Moderate	Not Related	Resolved without Sequelae
persistent ductus arteriosus	Oropharyngeal Surfactant	Moderate	Not Related	Ongoing
persistent ductus arteriosus	No Intervention	Mild	Not Related	Resolved without Sequelae
persistent ductus arteriosus	No Intervention	Moderate	Not Related	Ongoing
pneumonia	No Intervention	Mild	Not Related	Resolved without Sequelae
pneumonia	Oropharyngeal Surfactant	Moderate	Not Related	Resolved without Sequelae
pulmonary hypertension	Oropharyngeal Surfactant	Moderate	Not Related	Resolved without Sequelae
pulmonary hypertension	No Intervention	Moderate	Not Related	Resolved without Sequelae
sepsis	No Intervention	Moderate	Not Related	Resolved without Sequelae
sepsis	No Intervention	Moderate	Not Related	Resolved without Sequelae
sepsis	Oropharyngeal Surfactant	Moderate	Not Related	Resolved without Sequelae
sepsis	Oropharyngeal Surfactant	Moderate	Not Related	Resolved without Sequelae
SEVERE HYPOXEMIA/BRADYCARDIA REFRACTORY TO MASK VENTILATION	Oropharyngeal Surfactant	Severe	Not Related	Resolved without Sequelae
Staphylococcus epidermidis	No Intervention	Severe	Not Related	Resolved without Sequelae
Umbilical bleeding on central catheter	Oropharyngeal Surfactant	Moderate	Not Related	Resolved with Sequelae

© 2023 Murphy MC et al. *JAMA Pediatrics.*

eReference

1. Gray RJ. A Class of K-Sample Tests for Comparing the Cumulative Incidence of a Competing Risk. The Annals of Statistics, 16(3), 1141–1154. (1988).

© 2023 Murphy MC et al. *JAMA Pediatrics.*
