# Supplementary material for: Prophylactic Oropharyngeal Surfactant for Preterm Newborns at Birth: A Randomized Clinical Trial
Source: JAMA Pediatr. 2023 Dec 11;178(2):117–24. doi: 10.1001/jamapediatrics.2023.5082 (PMC10714282; doi:10.1001/jamapediatrics.2023.5082)
Supplement: Supplement 3. — Data Sharing Statement [file jamapediatr-e235082-s003.pdf]

Data Sharing Statement

Murphy. Prophylactic Oropharyngeal Surfactant for Preterm Newborns at Birth. *JAMA Pediatr*. Published November 27, 2023. doi:10.1001/jamapediatrics.2023.5082

Data
Data available: No

Additional Information
Explanation for why data not available: Available upon request
